# Combination of GC-IMS and Nano-LC/HRMS Reveals the Mechanism of Superheated Steam Glycosylation Modification in Improving Oyster Peptide Flavor

**DOI:** 10.3390/foods15020236

**Published:** 2026-01-09

**Authors:** Li-Hong Wang, Jun-Wei Zhang, Zong-Cai Tu, Xiao-Mei Sha, Yong-Yan Huang, Zi-Zi Hu

**Affiliations:** 1 National R&D Center for Freshwater Fish Processing, College of Life Science, Jiangxi Normal University, Nanchang 330022, China; 2 State Key Laboratory of Food Science and Resources, Nanchang University, Nanchang 330047, China

**Keywords:** oyster peptides, superheated steam, glycosylation, volatile compounds, deodorization

## Abstract

This study investigated the effect of superheated steam (SS) assisted glycosylation modification on the flavor profile of oyster peptides (OP), and explored the correlation between key flavor compounds and glycosylation degree using Gas Chromatography–Ion Mobility Spectrometry (GC-IMS) and nano-scale Liquid Chromatography coupled with High-Resolution Mass Spectrometry (nano-LC/HRMS). The results indicated that SS treatment accelerated the glycosylation process, reduced free amino groups level, and distinguished their unique flavor through E-nose. GC-IMS analysis detected 64 signal peaks including 13 aldehydes, 6 ketones, 7 esters, 6 alcohols, 2 acids, 2 furans and 5 other substances. And it was revealed that SS-mediated glycosylation treatment reduced the levels of fishy odorants like Heptanal and Nonanal, while promoting the pleasant-smelling alcohols and esters. In addition, Pearson correlation showed a positive correlation between excessive glycation and the increase in aldehydes, which might cause the recurrence of undesirable fishy notes. Further nano-LC/HRMS analysis revealed that arginine and lysine acted as the main sites for glycosylation modification. Notably, glycosylated peptides such as KAFGHENEALVRK, DSRAATSPGELGVTIEGPKE, generated by mild SS treatment could convert into ketones and pyrazines in subsequent reactions, thereby contributing to overall sensory enhancement. In conclusion, SS treatment at 110 °C for 1 min significantly improved the flavor quality of OP and sustains improvement in subsequent stages, providing theoretical support for flavor optimization of oyster peptides.

## 1. Introduction

Oysters, specifically the Pacific oyster *Crassostrea gigas* (Family: Ostreidae), are the most widely cultivated shellfish globally and are prevalent in China’s coastal regions. Known for their delicate meat and rich protein content, as well as their substantial vitamins and trace elements, oysters are often referred to as “Marine milk” [1]. In China, the most traditional methods of processing oysters include consuming fresh, drying into products, or using as seasonings, which generally results in a low overall utilization rate. Therefore, deep-processing oyster products have become a market focus. Enzymatic hydrolysis, which utilizes different types of proteases to break down protein raw materials, offers advantages such as mild conditions and ease of control [2,3], and has become one of the primary methods for producing oyster peptides (OP). Research has shown that OP derived from enzymatic hydrolysis exhibits antihypertensive, antioxidant, antitumor, antifatigue, and anticoagulant properties [4,5]. They can also promote sexual reproduction and improve the structure of intestinal flora [6].

However, oysters and their hydrolysates (OP) possess unpleasant fishy flavor substances such as short-chain aldehydes and unsaturated aldehydes [7], which limits the application fields of oysters and hinders their high-value utilization. To address this issue, researchers have developed methods for fishy flavor removal, which can be broadly divided into two categories. One approach removes the fishy flavor directly through adsorption by deodorants or encapsulation of volatile compounds. The other approach masks the fishy flavor by generating other aromatic substances, such as through the Maillard reaction, microbial fermentation, and spice marination [8]. The Maillard reaction is widely used to mask undesirable flavors and enhance food taste profiles. In the initial phase of this reaction, known as glycosylation, compounds including N-substituted glycosylamine and 1-amino-1-deoxy-2-ketose are produced. This stage involves the condensation of free amino groups found on protein side chains with the carbonyl groups present in reducing sugars [9]. These early products act as precursors to volatile flavors, continuously participating in the reaction and thereby affecting the food’s taste profile. Research conducted by Liu et al. [10] demonstrated that the level of glycosylation directly influenced the presence of volatile compounds, thereby enhancing the aromatic qualities of silver carp.

Superheated steam (SS), as an emerging and efficient thermal processing technology, demonstrates unique advantages in food processing. This technology involves heating saturated steam beyond its boiling point under pressurized conditions, utilizing combined heat transfer mechanisms such as convection, condensation, and radiation to achieve highly efficient processing [11]. Currently, SS has been widely applied in drying, sterilization, and quality improvement of fruits, vegetables, cereals, meat, and dairy products, showing remarkable effectiveness in reducing harmful substances and enhancing food quality [12]. Specifically, Wang et al. [13] demonstrated that compared to charcoal, infrared, and microwave heating, superheated steam treatment significantly inhibits lipid oxidation in fried beef patties, thereby reducing the formation of carcinogenic heterocyclic amines. Chindapan et al. [14] found that this technology enhances the flavor characteristics of Robusta coffee beans, expanding their commercial application prospects. Takemitsu et al. [15] also reported that superheated steam treatment can reduce off-flavor compounds such as aldehydes and acids in barley by approximately 50%, significantly improving its palatability.

The unique thermophysical properties of SS, such as its high thermal conductivity and continuous dry flow characteristics, demonstrate significant potential in driving the glycosylation process during the initial stage of the Maillard reaction. In promoting glycosylation, SS exerts synergistic effects through multiple mechanisms: its excellent thermal conductivity enables rapid and uniform heating of materials, providing sufficient activation energy for the condensation reaction between free amino groups in peptides and carbonyl groups in reducing sugars; meanwhile, the continuous dry flow effectively removes water molecules generated during the reaction, shifting the reaction equilibrium toward glycosylation products and thereby enhancing reaction rate [16]. According to Chen et al. [17] prolonged superheated steam treatment led to a significant increase in Maillard reaction products (MRP) within the β-lactoglobulin-glucose (βlg-Glu) system. Particularly in the flavor improvement of oyster peptides, this technology offers a dual advantage: generating desirable flavor compounds through glycosylation while simultaneously removing primary fishy odor components, achieving synergistic flavor enhancement [18,19]. Despite the considerable potential of superheated steam technology, systematic research on its application in glycosylation-mediated flavor modification of oyster peptides remains relatively limited.

To comprehensively elucidate the flavor transformation mechanism, advanced analytical techniques are required. On the one hand, the rapid and sensitive profiling of volatile organic compounds is crucial. Gas Chromatography-Ion Mobility Spectrometry (GC-IMS) has emerged as a powerful tool for visualizing food flavors, renowned for its high sensitivity, rapid analysis, and ability to detect volatile fingerprints without complex pre-treatment [20]. On the other hand, a deep understanding at the molecular level necessitates the precise identification of modified peptide segments. Nano-scale Liquid Chromatography coupled with High-Resolution Mass Spectrometry (nano-LC/HRMS), as a cornerstone of modern food proteomics, offers unparalleled sensitivity and accuracy for characterizing low-abundance glycated peptides in complex systems [21,22]. The combination of these two techniques provides a holistic strategy, linking macroscopic flavor changes to microscopic molecular modifications.

Therefore, this study explored the molecular mechanism underlying the deodorization of glycosylated OP mediated by SS. The extent of glycosylation of glycosylated OP was assessed by measuring its free amino group content. An E-nose, GC-IMS in conjunction with principal component analysis (PCA), and orthogonal partial least squares discriminant analysis (OPLS-DA) were used to assess the volatile components of the OP. The glycosylated peptide segments were also identified using nano-LC/HRMS, and the results provided deeper insights into how the glycosylation process influences the OP’s flavor.

## 2. Materials and Methods

### 2.1. Materials and Reagents

Fresh oysters were acquired from Rushan Breeding Base (Rushan, Shandong, China, October 2023). Neutrase (100,000 U/g) and Flavourzyme (30,000 U/g) were sourced from Beijing Solarbio Science and Technology Co., Ltd. (Beijing, China). Analytical-grade glucose and o-phthaldialdehyde were also supplied by the same company. Chromatographic-grade formic acid and acetonitrile (98%) were sourced from Beijing Merck Drugs and Biotechnology Co., Ltd. (Beijing, China).

### 2.2. Preparation of OP

The experiment was conducted with slight modifications based on Zhang et al. [20]. After shelling and removing the meat from fresh oysters, the meat was weighed and distilled water was added in a 1:2 ratio depending on the bulk of the meat to the liquid. Blended the oysters in a tissue homogenizer for 5 min. Subsequently, the pH of the oyster homogenate was adjusted to 7.0 using 0.1 M NaOH. Then, a composite enzyme mixture (Neutrase–Flavourzyme = 1:1, *m*/*m*) was added at a dosage of 0.7% (*m*/*m*, relative to oyster meat mass) to initiate enzymatic hydrolysis. The mixture was hydrolyzed enzymatically for four hours in a water bath at 50 °C. After that, the enzyme was heated for ten minutes at 100 °C in a water bath to render it inactive. In a refrigerated centrifuge set at 4 °C, the sample was centrifuged for 20 min at 8000 rpm to produce the OP solution. After that, the supernatant was gathered and freeze-dried for subsequent use.

### 2.3. Preparation of the OP-Glucose System

The freeze-dried OP powder was dissolved in deionized water, followed by addition of glucose at a 1:1 (*w*/*w*) ratio. After complete dissolution by vortex mixing, the OP-glucose solution was divided into 7 equal aliquots and freeze-dried for subsequent experiments.

### 2.4. Glycosylation of the OP-Glucose System

The superheated steam generator (ZKMB-28GB17, Vatti, Zhongshan, Guangdong, China) was preheated to the set temperature. Then, 0.5 g of freeze-dried OP-glucose mixture samples was weighed, placed in 20 mL glass vials, and subjected to heat treatment in the superheated steam chamber. The treatment temperatures were 110 °C and 130 °C, with durations of 1, 3, and 5 min, respectively. To stop the glycosylation reaction, all samples were quenched for ten minutes in an ice-water bath. These samples, treated under different conditions, were named 110-1, 110-3, 110-5, 130-1, 130-3, and 130-5. Additionally, an untreated OP group was set up as a comparison, named TAI. The freeze-dried OP-glucose mixture that has not undergone any further heat treatment was named as CON.

### 2.5. Determination of the Free Amino Groups

With a small modification, the o-phthalaldehyde (OPA) method for determining free amino groups was carried out as Huang et al. [23] reported. Lysine was used at values ranging from 0 to 0.60 mg/mL to create a standard curve. Dissolved the glycosylated OP samples in ultrapure water and diluted to 1 mg/mL. After mixing a 100 μL aliquot of 1 mg/mL material with 2 mL of o-phthalaldehyde, the absorbance at 340 nm was measured using a UV-visible spectrophotometer (U-2910, Hitachi, Tokyo, Japan). The standard curve equation was then used to determine the sample’s free amino groups content.

### 2.6. Determination of Volatile Flavor Profile by E-Nose

The smell of OP was examined using the E-nose (PEN3, Airsense, Schwerin, Germany). Following a slightly adapted version of the method outlined by Liu et al. [24], 0.5 g of the glycosylated OP samples was weighed, transferred to a 20 mL sample vial, and securely sealed. The vials were incubated in a 60 °C water bath for 30 min, followed by equilibration at room temperature before the PEN3 probe was inserted for measurement. The measurement parameters were as follows: 400 mL/min of carrier gas flow rate, 90 s for cleaning, 10 s for zeroing, 5 s for preparation, and 100 s for measurement. The E-nose features ten metal oxide sensors, each designed to detect specific types of compounds: W1C (aromatics), W5S (nitrogen oxides), W3C (aromatic, ammonia), W6S (hydrogen), W5C (short-chain alkanes, aromatics), W1S (methyl compounds), W1W (inorganic sulfides), W2S (alcohols, aldehydes, ketones), W2W (aromatics, organic sulfides), and W3S (long-chain alkanes).

### 2.7. Determination of Volatile Compounds by HS-GC-IMS

HS-GC-IMS (Flavour Spec, G.A.S, Dortmund, Germany) was utilized to examine the volatile chemicals in the samples. The method was carried out based on the work of Nie et al. [25] with minor modifications. Separately, dissolved 0.5 g of each sample in 8 mL of deionized water, then aspirated 2 mL of the mixture into a 20 mL headspace container. A 500 μL aliquot was injected for examination following a 15 min incubation period at 60 °C. For gas chromatography (GC), separation was achieved on an Agilent DB-WAX capillary column (30 m × 0.25 mm × 0.25 μm) maintained at a constant temperature of 60 °C. High-purity nitrogen (≥99.999%) served as the carrier gas, with its flow rate programmed as follows: 2 mL/min for 2 min, increased to 10 mL/min over 10 min, then to 100 mL/min over 20 min, and finally to 150 mL/min for the last 10 min. For ion mobility spectrometry (IMS), the drift tube (9.8 cm) was operated at 45 °C with a uniform electric field of 400 V/cm. High-purity nitrogen (≥99.999%) was used as the drift gas at a constant flow rate of 150 mL/min. The NIST database and the GC-IMS library standards were applied to qualitatively analyze the volatile compounds.

### 2.8. Sensory Evaluation Test

The sensory evaluation test was adapted with modifications from the approach described by Zhao et al. [26]. A panel of ten trained sensory evaluators (5 male, 5 female, aged 23–28) was assembled. This study focused specifically on odor attributes. Through preliminary analysis and discussion, the panel identified five key attributes for evaluation: “fishy odor”, “seafood odor”, “burnt aroma”, “caramel odor”, and “meaty aroma”, along with overall acceptability. A 10-point scale (0–9) was used for assessment, where scores of 0–2 indicated extremely weak intensity/extreme dislike, 3–5 represented moderate intensity/moderate liking, and 6–9 denoted extremely strong intensity/strong liking. All evaluations were conducted in a standard sensory laboratory under controlled conditions—ensuring a well-ventilated, odor-free, and quiet environment. Between samples, evaluators observed a 10 min rest interval to minimize carryover effects.

### 2.9. Identification of Glycated Peptides by Nano-LC/HRMS

Adapted from Fu et al. [22], the nano-LC/HRMS method for identifying glycosylation sites was performed with minor adjustments. The glycated oyster peptide samples were desalted using a C18 cartridge. The peptides were then lyophilized, reconstituted in 0.1% formic acid aqueous solution, and analyzed using an Orbitrap Fusion Tribrid mass spectrometer (Thermo Fisher) coupled to an EASY-nano LC 1200 system (Thermo Fisher Scientific, Waltham, MA, USA). A 5 μL sample was injected by the autosampler onto a loading column (Thermo Scientific EASY column, 100 μm × 2 cm, 5 μm, C18) and subsequently separated on an analytical column (Thermo Scientific EASY column, 75 μm × 10 cm, 3 μm, C18) at a flow rate of 300 nL/min. The mobile phase consisted of 0.1% formic acid in water (A) and 0.1% formic acid in 84% acetonitrile (B). A linear gradient was run from 0% to 35% B over 75 min, followed by an increase to 100% B over 7 min (75–82 min), and a final hold at 100% B for 3 min (82–85 min). The mass spectrometer was operated in a data-dependent mode, automatically switching between full-scan MS and MS/MS acquisitions. The detection was performed in positive ion mode (ESI+) with a spray voltage of 2.2 kV. Full-scan MS spectra (*m*/*z* 350–1550) were acquired in the Orbitrap mass analyzer at a resolution of 60,000, with an automatic gain control (AGC) target of 2 × 10^6^ and a maximum injection time of 50 ms. The MS/MS spectra were obtained at a resolution of 15,000, with a normalized collision energy of 32 eV and an underfill ratio of 0.1%. The raw files were submitted to the Sequest server via Proteome Discoverer (version 1.4.1.14) for database searching using ETD/HCD fragmentation modes. The database used for the search was uniprot_Crassostrea_gigas_83441_20230518. For ETD spectra, the precursor ion tolerance was set to 20 ppm, and the fragment ion tolerance was set to 1.2 Da. For HCD spectra, the precursor ion tolerance was set to 20 ppm, and the fragment ion tolerance was set to 0.1 Da. The result filtering parameters were as follows: FDR < 0.01. Variable modifications included: Oxidation (K, M, P) and C_6_H_10_O_5_ (K, R).

### 2.10. Statistical Analysis

Every experiment was carried out in triplicate, and the mean ± SD was used to express the results. Data were analyzed using SPSS Statistics 17.0, and significant differences between groups were compared using one-way ANOVA followed by Tukey’s test, with *p* < 0.05 indicating statistical significance. For GC-IMS analysis, the Reporter and Gallery Plot plug-ins were utilized to evaluate sample differences and generate fingerprints, while SIMCA 14.1 (MKS Umetrics AB Inc., Andover, MA, USA) was employed for multivariate statistical analysis of volatile compounds. The relationship between glycosylation degree and volatile compound content was assessed using Pearson correlation analysis in Origin Pro 2024 (Origin Lab Corp, Northampton, MA, USA).

## 3. Results

### 3.1. Analysis of the Free Amino Groups

**Figure 1:**
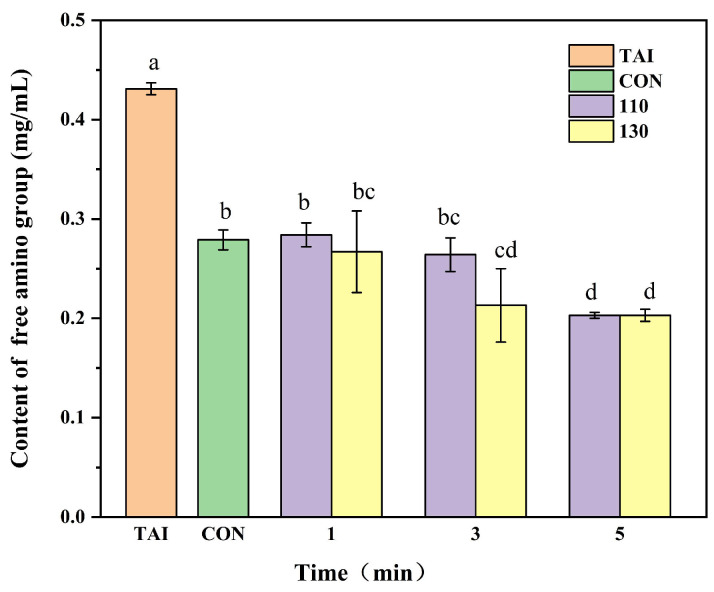
Impact of glycosylation reaction on the free amino group content of OP. TAI: unprocessed OP; CON: OP with added sugar but no SS reaction; 110: the temperature of SS is 110 °C; 130: the temperature of SS is 130 °C. Different letters in the figure indicate statistically significant differences among groups (*p* < 0.05).

A decrease in free amino groups content, a measure of the degree of glycosylation, results from the gradual reaction of proteins’ free amino groups with sugar carbonyl groups during glycosylation [27]. When glucose was added, the free amino groups content significantly decreased (*p* < 0.05) in comparison to the TAI group, as seen in Figure 1. The significant loss of lysine residues suggested that alterations in protein conformation might have occurred. Given the crucial role of lysine in maintaining β-sheet structure [28], its modification could have been a potential contributing factor to the reduction in ordered secondary structures. With higher SS temperatures and extended processing times, the free amino groups content generally decreased, aligning with the results reported by Zhang et al. [29]. The high thermal conductivity of SS provides sufficient activation energy for the condensation reaction between free amino groups and reducing sugars, increases the collision rate between protein and sugar molecules, and consequently intensifies the degree of glycosylation [30]. Intriguingly, the 110-1 group’s free amino groups content did not alter much compared to the CON group, possibly due to the low temperature and short duration of the SS treatment, which failed to instantly remove the deeply embedded moisture in the samples. Consequently, reducing sugars could not replace the spatial positions of water molecules for further glycosylation modification [31]. As the reaction time extended, the free amino groups content in the 110-5 group and the 130-5 group became essentially the same. This convergence may result from conjugate breakdown, structural alterations, and glycosylation site saturation, which decreased OP’s ability to bind glucose and prevented further reductions in free amino content [32].

### 3.2. Analysis of E-Nose

**Figure 2:**
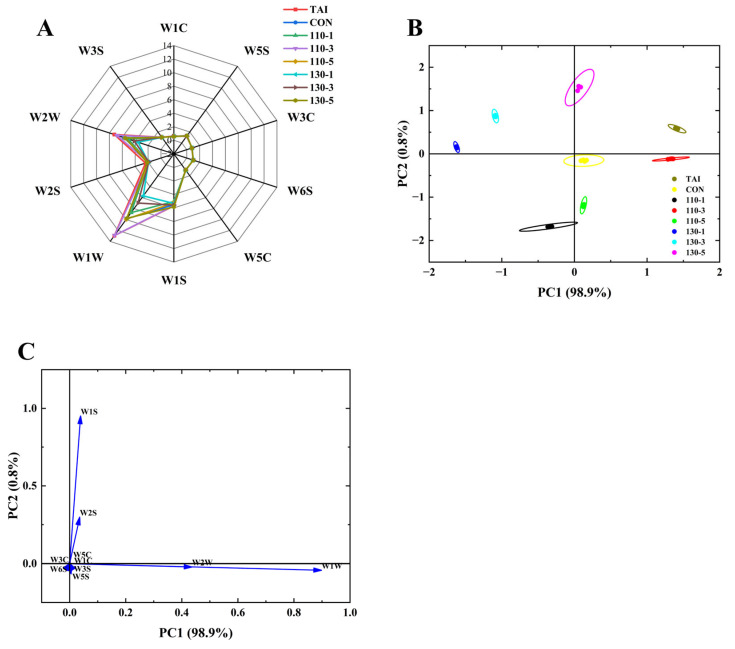
(**A**) Radar Chart; (**B**) PCA Plot; (**C**) Loading Plot of the E-nose. TAI: unprocessed OP; CON: OP with added sugar but no SS reaction; 110-1: SS temperature of 110 °C was treated for 1 min; 110-3: SS temperature of 110 °C was treated for 3 min; 110-5: SS temperature of 110 °C was treated for 5 min; 130-1: SS temperature of 130 °C was treated for 1 min; 130-3: SS temperature of 110 °C was treated for 3 min; 130-5: SS temperature of 130 °C was treated for 5 min.

The E-nose, which simulates biological olfactory functions through metal oxide sensors, is a highly efficient and rapid instrument for detecting the volatile flavor profiles [24]. As depicted in Figure 2A, the response values of six sensors, W3S, W1C, W5S, W3C, W6S, and W5C, remained relatively low both before and after SS treatment, with negligible variations observed across the samples. This suggested that the contents of nitrogen oxides, ammonia compounds, hydrides, and short-chain alkanes did not change significantly during the sample processing. Among the eight sample groups, the sensors W2W, W2S, W1W, and W1S demonstrated high sensing capabilities and notable group differences, indicating that the primary flavor chemicals in the OP were sulfides, aromatic compounds, alcohols, aldehydes, and ketones. Moreover, the samples’ response metrics on the W1W and W2W sensors varied considerably, suggesting that these two sensors were able to differentiate between OP samples that had been exposed to SS at various temperatures and times.

In order to facilitate efficient sample separation, Principal Component Analysis (PCA) is a technique used to reduce the dimensionality of data from E-nose sensors, extract important distinctive variables, and emphasize those with the highest contribution rates [33]. PCA centers on the responses of different sensors of the E-nose and can characterize the flavor differences between different samples. The farther the distance between the sample distribution areas, the greater the flavor differences. According to Figure 2B, PC1 accounted for 98.9% of the variance, and PC2 accounted for 0.8%, resulting in a cumulative variance of 99.7%, well above the threshold of 85%. This demonstrates that the two principal components effectively captured the variations in sample flavor profiles. Within the principal component analysis area, the TAI group and the 110-3 group overlapped on the first principal component, while the 130-5, 110-5, and 110-1 groups also overlapped with the CON group on the first principal component. However, significant differences were noted in the second principal component among these groups. Overall, the distinct separation of sample positions across groups demonstrates that the E-nose effectively distinguishes the main component variations in OP under different SS treatments. To further evaluate sensor contributions, the loading plot (Figure 2C) was analyzed. Sensors farther from the origin showed greater contributions The findings revealed that sensors W1W and W2W contributed more significantly to PC1, whereas W1S and W2S showed a higher contribution rate to PC2. This indicated that sensors W1W, W2W, W1S, and W2S were important in determining the flavor of OP samples, which was in line with the radar chart’s findings.

### 3.3. Analysis of Volatile Compounds in OP Treated Under Various SS Conditions

#### 3.3.1. Qualitative Analysis of Volatile Compounds in OP Treated Under Various SS Conditions

To better examine the variations in flavor characteristics of glycosylated OP induced by SS, GC-IMS was employed to separate the volatile compounds. The results (Table 1) revealed 64 signal peaks in all, encompassing monomers, dimers, and neutral compounds. These compounds comprise 13 aldehydes, 6 ketones, 7 esters, 6 alcohols, 2 acids, 2 furans, and 5 other substances, with the latter category including 3 unidentified compounds.

**Figure 3:**
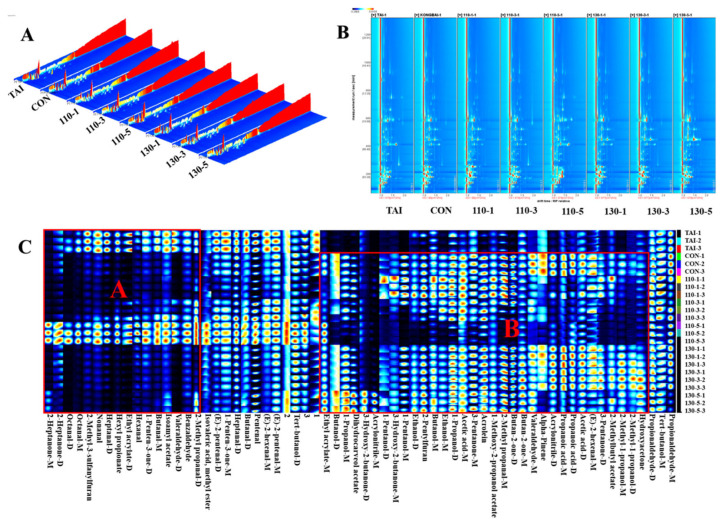
(**A**) Three-dimensional spectra of volatile compounds; (**B**) GC-IMS two-dimensional spectra of volatile compounds; (**C**) fingerprint of volatile compounds in different SS conditions of OP. The differential volatile compounds in oyster peptides, induced by glycosylation, are manifested as distinct areas A and B in the fingerprint.

Three-dimensional spectra (Figure 3A) illustrate the distribution of volatile compounds and their relative abundance in the sample set, where ion migration time (ms), retention time (s), and ion peak intensities are plotted on the x-, y-, and z-axes, respectively, in the three-dimensional spectra [34]. It was clear that there were significant variations in the locations and peak intensities of volatile chemicals in OP treated under various SS conditions, even if their distribution patterns were comparable. Figure 3B showed a two-dimensional spectrum obtained through dimensionality reduction techniques. The red vertical line at X-axis 1.0 represents the RIP (Reaction Ion Peak). The color intensity of the spots indicates the signal strength of certain volatile organic chemicals; darker colors indicate higher concentrations and stronger signals. Each point on either side of the RIP peak represents an identified volatile molecule [35]. The volatile compound compositions in samples treated with SS at different temperatures and durations formed points with varying positions and colors in the spectrum. The 130-5 and 110-5 groups exhibited significantly higher volatile compound abundance, likely resulting from enhanced glycosylation-driven flavor formation under prolonged SS exposure.

To correctly and graphically depict the differences in volatile flavor compounds between several sample groups, a fingerprint plot as shown in Figure 3C was constructed. In this plot, every row represented all volatile compounds in a sample group and every column represented the difference in content of the same compound among different sample groups, with darker colors indicating higher content. As shown in Figure 3C, the Propionaldehyde-M, Tert-butanol-M, Propionaldehyde-D, Tert-butanol-D, (E)-2-pentenal-M, (E)-2-hexenal-M, Pentenal, Butanal-D, Heptanal-D, 1-Penten-3-one-M, (E)-2-pentenal-D, Isovaleric acid, Methyl ester-M, were detected in all samples. These compounds possessed pungent, waxy, moldy, yeast-like, and intense oily flavor, collectively forming the basic aroma of the OP samples.

**Figure 4:**
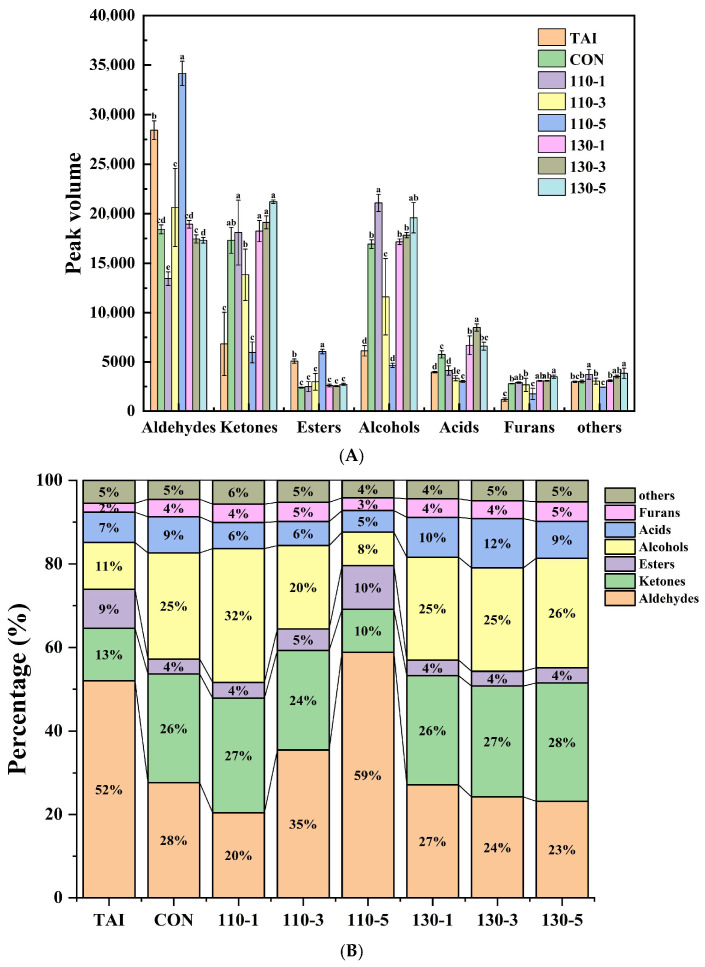
(**A**) Peak volumes of volatile compounds in different OP samples; (**B**) the percentage of various volatile compounds in different OP samples. Different letters in the figure indicate statistically significant differences among groups (*p* < 0.05).

In Area A, the TAI group exhibited high levels of nine aldehydes, including 2-Methyl propanal-D, Benzaldehyde, Valeraldehyde-D, Butanal-M, Hexanal, Heptanal-D, Nonanal, Octanal-M, Octanal-D. Aldehydes, typically originating from lipid oxidation, significantly influenced aroma owing to their low detection thresholds [18]. Studies have shown that low molecular weight aldehydes such as Heptanal, Nonanal, and Octanal possess strong fishy and oily flavor, serving as the primary compounds contributing to a fishy flavor [36]. After SS treatment, the content of aldehydes in the samples decreased significantly, with the 110-1 group showing the lowest content (Figure 4). Interestingly, the aldehydes content increased with the extension of reaction time at 110 °C, while it decreased with the extension of reaction time at 130 °C. This was mainly because: at 110 °C, oyster peptides underwent peptide bond cleavage and amino acid pyrolysis, while promoting the oxidation of unsaturated fatty acid side chains, and more aldehydes were generated as the reaction time prolongs; at the higher temperature of 130 °C, the degradation of aldehydes was accelerated, and aldehydes reacted with free amino groups in the initial stage of Maillard reaction to form Schiff bases [10,37]. At this time, the degradation rate of aldehydes was greater than the generation rate, which resulted in a decrease in their content.

Area B represented volatile compounds produced after glycosylation treatment with SS. As the glycosylation reaction progresses with SS, more alcohols, ketones, acids, and furans were generated. In the glycosylation system, alcohols, ketones, and acids typically had higher flavor thresholds and did not significantly impact sensory properties [38]. Among these, the 110-1 group had the highest alcohols content. Alcohols, mainly resulting from lipid oxidation and decomposition, possess pleasant fruity and floral aromas [39,40]. Like aldehydes, ketones are linked to lipid oxidation and play a significant role as flavor precursors in reaction known as the Maillard system [41]. Both alcohols and ketones experienced different degrees of change during the glycosylation process, indicating their involvement in the glycosylation reaction and the generation of additional volatile compounds. Acids, often produced by the breakdown of long-chain fatty acids, contributed minimally to flavor due to their high thresholds and low concentrations. Furan, formed through carbohydrate dehydration, fatty acid oxidation, or the Amadori rearrangement mechanism, was predominantly detected in glycosylated samples. Notably, 2-Pentylfuran, with its low threshold, imparted a pleasant aroma characterized by sweetness and roasted notes [24]. Overall, the 110-1 group exhibited the best deodorizing effect, as it had the lowest aldehydes content and the highest alcohols content. The mild SS treatment (110-1 group) provided sufficient thermal energy to drive the initial glycosylation reaction while effectively preventing the reaction from progressing to advanced stages or triggering excessive degradation. This condition not only facilitated the participation of original fishy aldehydes in the reaction but also suppressed the generation of new aldehydes. In contrast, more intense or prolonged treatments were associated with an increase in the concentration of specific aldehydes. We hypothesize that these conditions may have disrupted the balance of initial reaction pathways, potentially promoting either the oxidative regeneration of carbonyls or progression into advanced Maillard stages that yield less desirable flavors. This indicates that mild SS-mediated glycation reactions are more effective in improving the undesirable flavor of OP.

#### 3.3.2. Multivariate Analysis of Volatile Compounds in OP Treated Under Various SS Conditions

**Figure 5:**
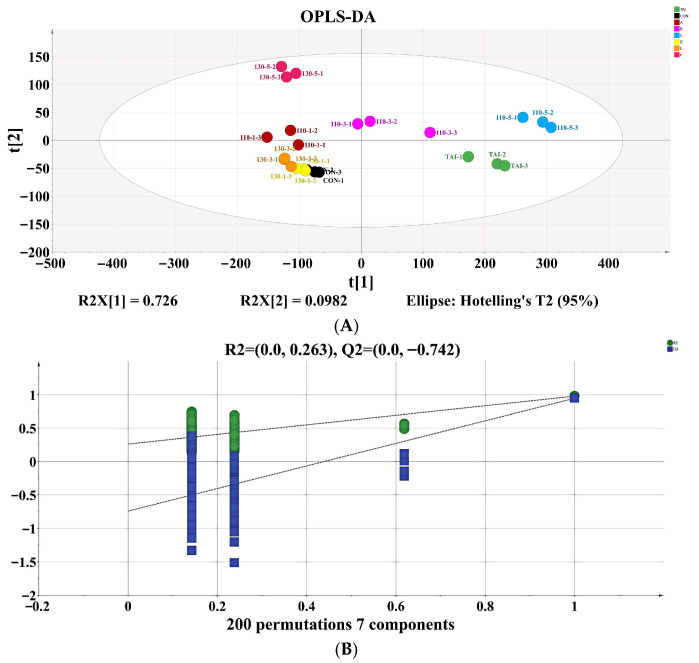

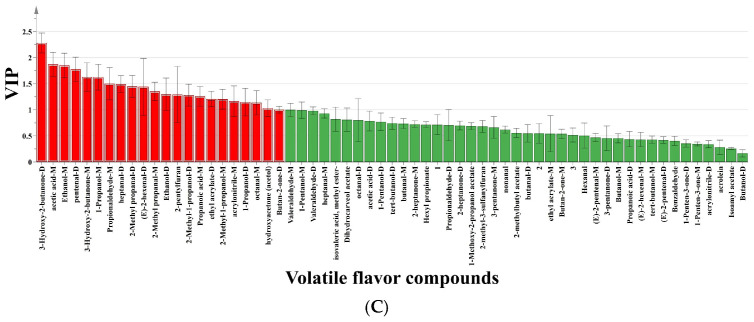
(**A**) OPLS-DA; (**B**) model cross-validation diagram; (**C**) VIP score of OPLS-DA model. In Figure 5C, the shading in red color indicates the flavor compounds that have VIPs > 1.0, and the shading in green color indicates the flavor compounds that have VIPs < 1.0.

Account on the volatile compound data collected by GC-IMS, we constructed an OPLS-DA model to comprehensively evaluate the flavor distinction in OP subjected to different SS treatments. The discriminant effect of the model was shown in Figure 5A, where the R^2^X, R^2^Y, Q^2^ value were 0.975, 0.865, and 0.652, respectively. These values demonstrated the model’s excellent reliability and its ability to predict the flavor of OP handled under various SS conditions. The 130-3 group, 130-1 group, and CON group were relatively close, suggesting that the volatile compounds in these three groups were similar. The other five groups were more dispersed, indicating major variations in the similarity of volatile compounds during the processing of OP with different SS treatments. Following 200 permutation tests, the model yielded R^2^ = 0.263 and Q^2^ = -0.742, with R^2^ exceeding Q^2^ and Q^2^ intersecting the negative y-axis (Figure 5B), confirming the absence of overfitting [42]. To better identify the key flavor driving the variances in OP treated with different SS situations, a VIP value plot was created. The higher the VIP value, the more that variable contributes to group distinction when using VIP > 1.0 as the criterion. Volatile substances with VIP > 1.0 were marked in red in Figure 5C. Using the thresholds of *p* < 0.05 and VIP > 1.0, 22 substances with significant contributions to OP samples were screened out. The differential contribution degrees, from largest to smallest, are 3-Hydroxy-2-butanone-D, Acetic acid-M, Ethanol-M, Pentenal, 3-Hydroxy-2-butanone-M, 1-Propanol-M, Propionaldehyde-M, Heptanal-D, 2-Methyl propanal-D, (E)-2-hexenal-D, 2-Methyl propanal-M, Ethanol-D, 2-Pentylfuran, 2-Methyl-1-propanol-D, Propanoic acid-M, Ethyl acrylate-D, 2-Methyl-1-propanol-M, Acrylonitrile-M, 1-Propanol-D, Octanal-M, Hydroxyacetone, and Butan-2-one-D. The results indicate that these volatile compounds could be used to distinguish OP samples treated under different SS conditions.

### 3.4. Analysis of Sensory Evaluation

**Figure 6:**
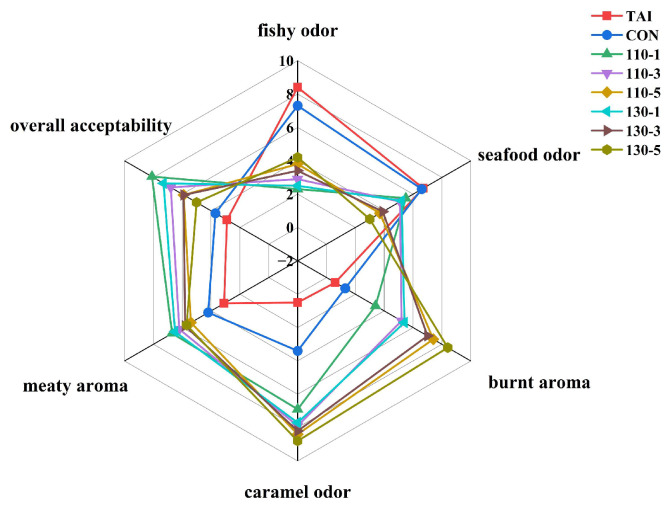
Sensory evaluation results of oyster peptides treated with different superheated steam.

The radar chart based on the sensory evaluation results was shown in Figure 6. The assessment covered six attributes: fishy odor, seafood odor, burnt aroma, caramel odor, meaty aroma, and overall acceptability. In terms of fishy odor, 110-1 and 130-1 groups received lower scores, indicating a milder fishy odor and better deodorization effect. For seafood odor, all groups treated with SS-assisted glycosylation showed reduced scores, with 130-5 group scoring the lowest. Regarding meaty aroma, the TAI group scored the lowest, followed by the CON group, which suggested that superheated steam-assisted glycosylation effectively enhanced the meaty aroma. As for burnt aroma and caramel odor, 130-5 group scored the highest, followed by 110-5 group, indicating that prolonged SS treatment led to excessive glycosylation in OP samples, thus intensifying the burnt aroma and caramel odor, which to some extent negatively affected the overall odor acceptability. Consequently, in terms of overall odor acceptability, 110-1 group was consistently rated as having the most desirable odor profile.

### 3.5. Analysis of Glycosylated Peptides

In order to unequivocally identify glycosylation sites, glycopeptides were subjected to Electron Transfer Dissociation (ETD) analysis, yielding high-quality tandem mass spectrometry data. As illustrated in the spectra provided in the Figures S1–S7, the ETD spectra typically exhibit strong signals and well-covered c- and z-ion series. This high-quality fragment information enabled reliable elucidation of the peptide sequences and, based on the mass differences between adjacent fragment ions, allowed for confident and precise localization of the glycosylation sites. For instance, in the case of the glycopeptide DVIDTNKDRTIDE identified in the CON group (Figure S1B), the mass difference between the adjacent c_6_ and c_7_ ions was determined to be 290.1449 Da. This observed mass shift significantly exceeds the theoretical residue mass of a lysine. Further calculation confirmed that this difference corresponded precisely to the sum of the lysine residue mass (128.09496 Da) and the glycan moiety mass (162.04994 Da), thereby confirming this site as a glycosylation site. Table 2 summarized the key data of the detected glycopeptides, including their peptide sequences, mass-to-charge ratios (*m*/*z*), charge, and mass deviations (ΔM). The mass deviations for all glycopeptides were within ±5 ppm, confirming the excellent agreement between our high-precision mass spectrometry measurements and the theoretical models. Overall, Lys (K) and Arg (R) are the main alteration sites, which is consistent with earlier findings suggesting that both of those amino acids may act as glycosylation sites in peptides produced from food [43]. Notably, the quantity of glycosylated peptides found rose in tandem with the temperature and length of the SS treatment, matching the level of glycosylation in the OP. Moreover, the three glycosylated peptides KAFGHENEALVRK, DVIDTNKDRTIDE and DSRAATSPGELGVTIEGPKE were mainly produced by slight glycosylation reactions, while GQRGIPGERGRDGDRGSNG, YISLEELYKIMTTK and SLYNKENKHVPLK were unique glycosylated peptides in group 130-5, suggesting that SS treatment might have exhibited selectivity for certain protein degradation pathways and saccharification reactions. This selectivity could have promoted the glycosylation reaction, possibly by exposing specific amino acid residues that are prone to modification. Furthermore, the glycosylated peptide DKDGKGKIPEEY was consistently present across different SS conditions, indicating that the specific amino acid (lysine) within this peptide might have possessed high reactivity. The flavor contribution was evaluated by the amino acid makeup and the protein of origin of the saccharide peptides [44]. Glycosylated peptides rich in lysine (K) and arginine (R) (such as DKDGKGKIPEEY and RRGESGPNGEPGRTGPPGPRGPRG) were efficient substrates for the Maillard reaction. The reaction rate between the ε-amino group of lysine or the guanidino group of arginine and the glycosyl group is generally fast in model systems, which might have facilitated the formation of Amadori rearrangement products (ARPs) under our experimental conditions. According to a common Maillard pathway [45], ARPs can undergo dehydration to form 3-deoxyglucosone (3-DG), which may further react to form furan rings. Subsequent oxidative modification of these furan rings, potentially involving interactions with amino groups and reactive oxygen species, provided a plausible route for the generation of the mild fruity aroma compounds that were detected. Furthermore, the detection of flavor substances with roasted and nutty aromas may be associated with another set of identified peptides, particularly those rich in hydrophobic amino acids (such as DSRAATSPGELGVTIEGPKE and SPFKVEVGPAKT). The theory that hydrophobic amino acid side chains could be oxidized by hydroxyl radicals to form alkoxyl radicals, followed by β-scission reactions resulting in C–C bond cleavage [46], provided a reasonable explanation for the formation of roasted-nutty compounds. Most of these glycosylated peptides were produced by slight glycosylation reactions. Flavor precursor proteins play a key role in altering flavor. The glycosylated peptides identified by different SS conditions were mainly released from the following proteins: EF-hand domain-containing protein, Sarcoplasmic calcium-binding protein, Filamin-C, Collagen α-2(I) chain, Myosin heavy chain, and striated muscle. Zhang et al. [47] identified oyster umami peptides and found myosin as the main precursor protein of potential umami peptides. According to Hu et al. [48], myosin contained a significant amount of savory chemicals, while collagen from all fish samples primarily contained sweet amino acids and bitter peptides As a result, myosin and collagen might be good protein sources for producing a lot of taste-active peptides.

### 3.6. Analysis of the Correlation Between Important Volatile Compounds and Glycosylation Degree

**Figure 7:**
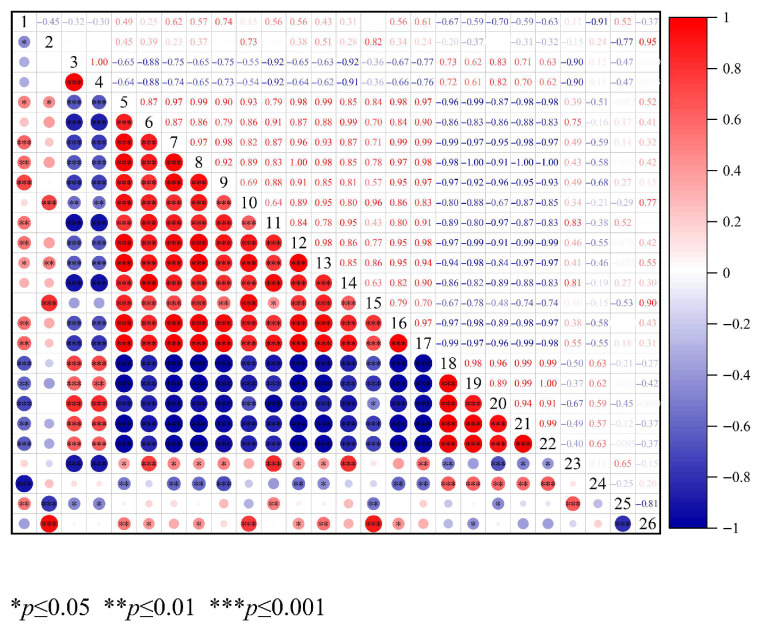
Correlation analysis between glycosylation degree and key volatile compounds. 1 represents free amino groups content; 2 represents the number of glycosylated peptides; 3 represents the response values of W1W; 4 represents the response values of W2W; 5-26 represent the contents of 3-Hydroxy-2-butanone-D, Acetic acid-M, Ethanol-M, 3-Hydroxy-2-butanone-M, 1-Propanol-M, (E)-2-hexenal-D, 2-Methyl propanal-M, Ethanol-D, 2-Pentylfuran, Propanoic acid-M, 2-Methyl-1-propanol-M, Acrylonitrile-M, 1-Propanol-D, Pentenal, Heptanal-D, 2-Methyl propanal-D, Ethyl acrylate-D, Octanal-M, Butan-2-one-D, Hydroxyacetone, Propionaldehyde-M, and 2-Methyl-1-propanol-D.

To systematically evaluate the glycosylation process of OP, free amino groups content and the number of glycosylated peptides were adopted as key evaluation metrics. The number of glycosylated peptides not only comprehensively reflected the diversity of glycosylation modification sites but also effectively mitigated quantitative biases caused by differences in ionization efficiency among glycosylated segments in nano-LC/HRMS technology [49,50]. This provided a more robust systematic basis for assessing glycosylation levels. For flavor characterization, the response signals from two characteristic sensors in the electronic nose were integrated with 22 key volatile compounds (VIP > 1) screened by GC-IMS. A correlation model between glycation indicators and flavor characteristics was systematically constructed using Pearson correlation analysis. Figure 7 displayed the findings. The quantity of glycosylated peptides, Pentenal, Heptanal-D, 2-Methyl propanal-D, Octanal-M, and Hydroxyacetone were significantly correlated negatively with the free amino group content (r = −0.91 to −0.45, *p* < 0.05). Conversely, significant positive associations were observed with 1-Propanol-M, 1-Propanol-D, and Ethanol-M (r = 0.65 to 0.79, *p* < 0.05). This indicated that as the free amino group content decreases, the number of glycosylated peptides continuously increases, and the glycosylation degree of OP intensifies. Meanwhile, substances such as Pentenal, Heptanal-D, 2-Methyl propanal-D, Octanal-M, and Hydroxyacetone increase to varied degrees. This implied that the intensification of glycosylation tends to encourage lipid oxidation, leading to the production of more aldehyde compounds, which is line with the studies of Khan et al. [51]. Pentenal, Heptanal-D, 2-Methyl propanal-D, and Octanal-M showed a positive correlation with the response values of W1W and W2W sensors (sensitive to sulfides and aromatic compounds) in the electronic nose. This was mainly because amino acids in oyster peptides (such as cysteine, phenylalanine, leucine) served as common precursors for sulfides, aromatic compounds, and aldehydes [52]. Superheated steam glycosylation accelerated the catabolism of these amino acids, which led to a simultaneous increase in the production of the three types of substances, thereby resulting in a positive correlation between the sensor response values and aldehyde content. However, these substances showed a negative correlation with 3-Hydroxy-2-butanone-D, Acetic acid-M, Ethanol-M, 1-Propanol-M, Ethanol-D, 2-Pentyl furan, 2-Methyl-1-propanol-M and 1-Propanol-D (r = −0.99 ~ −0.46, *p* < 0.05). This indicated that alcohols and ketones participate in glycosylation reactions, leading to creating more ephemeral flavor molecules. Complex connections were shown between the degree of glycosylation and the concentration of important volatile chemicals. The mutual transformation among alcohols, ketones, acids, and aldehydes results in the production of a richer variety of volatile aromatic substances [10].

## 4. Discussion

This study systematically investigated how SS conditions regulate the flavor profile of OP through glycosylation. The results demonstrated that SS treatment significantly accelerated the glycosylation rate of OP, as evidenced by a notable reduction in free amino group content. This structural modification was directly correlated with marked changes in volatile compounds. Specifically, the content of key fishy odorants such as heptanal, nonanal, and octanal was significantly reduced, effectively mitigating the characteristic marine off-flavor. Concurrently, SS-assisted glycosylation promoted the formation of various flavor-active compounds, including alcohols, ketones, acids, and esters, thereby enriching the overall aromatic complexity. However, correlation analysis and sensory evaluation results revealed a critical finding: excessive reaction intensity was associated with the accumulation of certain aldehydes, ultimately negatively affecting sensory quality, whereas moderate glycosylation (110-1) effectively enhanced flavor acceptability. This nonlinear relationship underscores the importance of optimizing SS parameters to achieve balanced flavor enhancement. To further elucidate the molecular basis of these changes, we identified 56 unique glycated peptides in different SS-treated samples using nano-LC/HRMS. These modified peptides, derived from various oyster protein precursors, provide direct molecular evidence of the glycosylation reaction. More importantly, as potential flavor carriers or precursors, their formation and distribution are fundamentally linked to the observed macroscopic flavor transformations. In conclusion, this work confirmed that controlled glycosylation mediated by superheated steam was an effective strategy for improving the flavor of oyster peptides. Our integrated analytical approach, combining volatile compound profiling with precise molecular modification data, not only provides a theoretical foundation for the practical application of OP but also establishes a robust methodological framework for the rational flavor design of peptide-based functional foods.

## Figures and Tables

**Table 1:** Volatile flavor compounds in different SS conditions of OP by GC-IMS.

Serial Number	Compound	CAS	Molecular Formula	RI	Rt (s)	Dt (ms)	Peak Volume							
							TAI	CON	110-1	110-3	110-5	130-1	130-3	130-5
	Aldehydes													
1	Acrolein	107-02-8	C3H4O	854.7	150.12	1.06158	96.14 ± 7.91b	128.92 ± 6.11ab	99.91 ± 16.66b	96.97 ± 22.03b	37.6 ± 2.15c	145.61 ± 13.88a	120.05 ± 8.09ab	52.85 ± 11.93c
2	Propionaldehyde-M	123-38-6	C3H6O	791.2	128.643	1.06521	2809.17 ± 20.74ab	3472.55 ± 114.67a	2031.42 ± 672.08b	2289.43 ± 876.15b	2188.94 ± 19.17b	3134.81 ± 100.92ab	2637.51 ± 347.55ab	931.53 ± 33.45c
3	Propionaldehyde-D	123-38-6	C3H6O	791.2	128.643	1.14514	810.38 ± 0.65c	850.94 ± 17.19bc	1004.88 ± 52.02a	863.76 ± 79.71bc	839.02 ± 3.57c	942.09 ± 7.89ab	1004.75 ± 43.18a	792.93 ± 36.06c
4	2-Methyl propanal-M	78-84-2	C4H8O	835.6	143.31	1.11818	1382.86 ± 12.69d	3059.57 ± 114.09a	2021.36 ± 493.1b	1450.88 ± 62.13cd	1061.79 ± 7.74d	3402.9 ± 74.42a	3198.57 ± 88.87a	1890.39 ± 217.33bc
5	2-Methyl propanal-D	78-84-2	C4H8O	828.9	140.98	1.28716	1908.11 ± 18.05b	470.45 ± 36.7c	758.84 ± 402.23c	2249.14 ± 66.72b	3726.65 ± 18.54a	331.73 ± 13.29c	461.68 ± 4.63c	2204.93 ± 445.33b
6	Butanal-M	123-72-8	C4H8O	879.6	159.482	1.27117	1085.48 ± 10.34a	226.55 ± 16.19c	129.84 ± 7.2c	568.89 ± 340.19b	1318.51 ± 29.43a	223.47 ± 15.07c	198.35 ± 11.56c	194.9 ± 47.05c
7	Butanal-D	123-72-8	C4H8O	879.2	159.299	1.10982	1074.54 ± 3.88a	620.99 ± 19.53c	536.69 ± 4.57c	900.86 ± 165.19b	1095.79 ± 12.11a	647.96 ± 17.44c	613.24 ± 5.76c	633.23 ± 80.76c
8	(E)-2-pentenal-M	1576-87-0	C5H8O	1143.8	347.067	1.10225	628.71 ± 10.23a	532.89 ± 25.02b	366.61 ± 41.58d	657.23 ± 55.23a	666.2 ± 9.77a	534.14 ± 46.57b	437.78 ± 29.82cd	466.06 ± 4.34bc
9	(E)-2-pentenal-D	1576-87-0	C5H8O	1142.2	345.155	1.35306	356.59 ± 5.57a	206.93 ± 25.99c	72.32 ± 16.52e	277.57 ± 55.72b	330.04 ± 24.16ab	181.81 ± 27.42cd	117.91 ± 14.4de	144.01 ± 7.71cd
10	Valeraldehyde-M	110-62-3	C5H10O	947.1	187.855	1.17166	399.18 ± 5.97d	1587.35 ± 62.12ab	1131.81 ± 409.85bc	912.9 ± 385.47cd	602.85 ± 12.72cd	1722.67 ± 39.99a	1616.15 ± 77.1ab	835.89 ± 116.3cd
11	Valeraldehyde-D	110-62-3	C5H10O	931.7	180.982	1.40295	1738.28 ± 74.44a	63.64 ± 8.15c	55.46 ± 1.88c	749.59 ± 468.21b	1601.05 ± 54.25a	80.02 ± 11.41c	61.13 ± 10.72c	43.47 ± 6.39c
12	(E)-2-hexenal-M	6728-26-3	C6H10O	1227	464.355	1.1776	432.4 ± 6.24a	224.78 ± 14.42c	155 ± 26.18d	353.9 ± 28.38b	364.7 ± 15.04b	172.88 ± 12.17d	145.56 ± 11.78de	110.45 ± 11.36e
13	(E)-2-hexenal-D	6728-26-3	C6H10O	1220.2	453.593	1.49464	164.93 ± 24.27d	1756.88 ± 40.8bc	1682.97 ± 43.45bc	1450.6 ± 701.72c	378.13 ± 234.15d	2228.37 ± 104.14b	2191.43 ± 102.11b	3955.01 ± 68.25a
14	Hexanal	66-25-1	C6H12O	1098.3	295.431	1.27601	294.02 ± 88.52b	115.56 ± 7.39c	109.85 ± 6.24c	214.38 ± 101.7bc	544.25 ± 67.05a	191.61 ± 18.25bc	137.07 ± 33.93c	140.28 ± 6.42c
15	Benzaldehyde	100-52-7	C7H6O	1491.6	947.48	1.14761	397.35 ± 15.25b	180.96 ± 10.5d	124.75 ± 19.25d	299.44 ± 64.34c	501.78 ± 7.29a	177.02 ± 14.87d	156.51 ± 4.1d	126.73 ± 44.72d
16	Pentenal	110-62-3	C5H10O	1001.7	215.236	1.43447	5434.21 ± 82.49b	2137.09 ± 75.72e	1427.62 ± 324.06e	4342.86 ± 748.41c	8640.6 ± 32.62a	2235.91 ± 153.74e	2029.81 ± 13.91e	3145.69 ± 559.63d
17	Heptanal-D	111-71-7	C7H14O	1193.2	413.564	1.69636	3883.4 ± 48.42a	681.14 ± 30.48b	295.59 ± 44.15b	723.89 ± 567.96b	3355.88 ± 354.32a	582.45 ± 42.96b	487 ± 27.45b	234.04 ± 9.81b
18	Heptanal-M	111-71-7	C7H14O	1195.7	417.036	1.33659	2618.87 ± 23.2a	1507.67 ± 12.2b	1021.71 ± 80.96c	1540.46 ± 513.6b	2600.19 ± 56.2a	1458.1 ± 52.78bc	1354.39 ± 51.26bc	1004.49 ± 19.41c
19	Octanal-M	124-13-0	C8H16O	1298.4	590.125	1.41441	1667.26 ± 543.81b	326.8 ± 10.75c	238.86 ± 38.7c	399.53 ± 189.23c	2537.5 ± 437.37a	298.03 ± 40.16c	276.89 ± 12.71c	262.19 ± 9.8c
20	Octanal-D	124-13-0	C8H16O	1298.4	590.125	1.80971	455.8 ± 235.24b	47.54 ± 3.75b	39.64 ± 2.97b	50.97 ± 10.94b	1025.12 ± 368.14a	48.4 ± 6.53b	41.78 ± 4.08b	36.05 ± 3.5b
21	Nonanal	124-19-6	C9H18O	1398.2	753.72	1.47845	779.56 ± 14.21a	219.24 ± 16.44b	151.21 ± 5.53b	236.28 ± 131.16b	740.65 ± 57.41a	193.42 ± 27.25b	162.41 ± 13.25b	114.77 ± 9.69b
	Ketones													
22	Hydroxyacetone	116-09-6	C3H6O2	1318.7	620.165	1.22002	204.22 ± 18.12d	821.75 ± 38.56b	363.5 ± 85.3c	464.93 ± 42.73c	1054.71 ± 7.59a	764.68 ± 65.66b	805.55 ± 20.16b	1046.44 ± 13.53a
23	3-Hydroxy-2-butanone-M	513-86-0	C4H8O2	1308.2	604.5	1.07428	2698.27 ± 1196.7b	5559.01 ± 224.57a	5869.39 ± 438.86a	5236.69 ± 685.37a	1153.48 ± 734.15c	5833.94 ± 237.59a	6106.35 ± 95.19a	6293.65 ± 57.28a
24	3-Hydroxy-2-butanone-D	513-86-0	C4H8O2	1298	589.5	1.33168	2319.95 ± 2002.3c	7938.6 ± 955.74ab	10,230.01 ± 2625ab	6423.65 ± 1917.64b	681.89 ± 405.83c	8619.83 ± 776.47ab	9540.47 ± 685.75ab	11,549.86 ± 210.32a
25	1-Penten-3-one-M	1629-58-9	C5H8O	1038.4	242.699	1.07726	327.76 ± 3.83a	186.96 ± 9.31b	112.32 ± 29.31c	223.52 ± 55.8b	282.14 ± 22.34a	197.09 ± 19.49b	158.21 ± 8.43bc	121.04 ± 12.36c
26	1-Penten-3-one-D	1629-58-9	C5H8O	1038.9	243.133	1.3101	231.47 ± 3.89b	81.73 ± 3.28cd	59.29 ± 3.12cd	98.72 ± 30.09c	301.87 ± 38.76a	108.95 ± 15.81c	77.26 ± 8.81cd	43.34 ± 5.06d
27	Butan-2-one-M	78-93-3	C4H8O	908.3	170.963	1.05616	293.53 ± 5.83cd	554.69 ± 8.52a	409.98 ± 117.47b	300.59 ± 33.79cd	265.07 ± 1.7d	572.52 ± 5.27a	526.36 ± 12.36a	386.64 ± 13.24bc
28	Butan-2-one-D	78-93-3	C4H8O	910.9	172.082	1.23714	276.3 ± 7.89c	1057.46 ± 77.01a	364.33 ± 162.01c	363.71 ± 30.35c	676.7 ± 8.87b	1123.09 ± 49.63a	1013.36 ± 29.47a	654.71 ± 6.82b
29	3-Pentanone-M	96-22-0	C5H10O	1000.7	214.536	1.11003	124.94 ± 0.74d	642.62 ± 36.13a	402.74 ± 14.98c	476.81 ± 86.89bc	93.49 ± 13.12d	561.81 ± 37.92ab	483.5 ± 23.29bc	539.41 ± 27.36b
30	3-Pentanone-D	96-22-0	C5H10O	998.3	212.814	1.3529	22.11 ± 1.17d	212.98 ± 9.8a	155.4 ± 2.94b	96.99 ± 43.81c	29.65 ± 2.27d	162.45 ± 7.82b	150.33 ± 1.4b	212.54 ± 22.68a
31	2-Heptanone-M	110-43-0	C7H14O	1186.3	403.581	1.22687	62.88 ± 3.29c	57.44 ± 4.34cd	45.5 ± 2.77e	57.99 ± 1.85cd	651.38 ± 9.53a	54.02 ± 3.13cd	56.47 ± 4.63cd	265.1 ± 5.57b
32	2-Heptanone-D	110-43-0	C7H14O	1189.6	408.355	1.64419	273.63 ± 4.05b	202.15 ± 5.92b	85.18 ± 9.38c	102.84 ± 47.59c	772.73 ± 39.33a	249.33 ± 57.8b	208.18 ± 3.74b	101.3 ± 9.32c
	Esters													
33	Ethyl acrylate-M	140-88-5	C5H8O2	1012.1	222.703	1.1277	45.99 ± 1.28c	175.43 ± 14.39bc	420.4 ± 148.66a	83.01 ± 20.42bc	35.83 ± 1.32c	208.5 ± 16.43bc	270.28 ± 17.92ab	249.29 ± 130.35ab
34	Ethyl acrylate-D	140-88-5	C5H8O2	1004.6	217.27	1.40542	2596.96 ± 32.21a	150.37 ± 7.47c	129.18 ± 13.65c	1047.46 ± 675.11b	2923.56 ± 156.87a	190.21 ± 20.74c	143.32 ± 14.19c	107.21 ± 27.91c
35	Isovaleric acid, methyl ester	556-24-1	C6H12O2	1027.4	234.113	1.17501	614.79 ± 7.79b	382.68 ± 16.79c	362.39 ± 176.63c	419.97 ± 77.34c	1195.75 ± 15.71a	505.13 ± 23.97bc	441.72 ± 25.93bc	315.26 ± 79.13c
36	1-Methoxy-2-propanol acetate	108-65-6	C6H12O3	1227.4	464.905	1.13358	87.09 ± 1.43d	539.15 ± 10.2a	233.44 ± 136.9bc	128.58 ± 18.03cd	219.27 ± 6.18bc	572.22 ± 3.2a	562.89 ± 3.77a	325.39 ± 12.89b
37	Isoamyl acetate	123-92-2	C7H14O2	1140.5	343.122	1.29436	176.76 ± 3.36a	77.95 ± 4.81bc	55.17 ± 15.49c	108.76 ± 48.24b	190.07 ± 7.35a	64.27 ± 4.57bc	52.4 ± 1.17c	66.22 ± 9.73bc
38	2-Methylbutyl acetate	624-41-9	C7H14O2	1131.1	331.849	1.30674	89.51 ± 3.22bc	72.85 ± 11.41bc	67.41 ± 20.57bc	83.3 ± 6.68bc	99.41 ± 3.42b	58.85 ± 3.04c	68.32 ± 3.25bc	331.25 ± 29.66a
39	Hexyl propionate	2445-76-3	C9H18O2	1346	663.142	1.42876	1066.4 ± 34.34a	207.31 ± 4.05c	147.67 ± 42.7c	488.77 ± 273.04b	1213.44 ± 78.97a	162.08 ± 5.48c	137.54 ± 1.58c	149.89 ± 8.89c
40	Dihydrocarveol acetate	20777-49-5	C12H20O2	1296.7	587.625	1.23423	400.91 ± 254.05cd	786.92 ± 19.56abc	1069.45 ± 344.28ab	623.51 ± 167.24bc	178.57 ± 15.47d	855.55 ± 112.08ab	873.99 ± 84.15ab	1181.33 ± 4.48a
	Alcohols													
41	Ethanol-M	64-17-5	C2H6O	969.2	198.254	1.12975	1481.08 ± 270.27cd	4582.52 ± 340.63b	6660.37 ± 628.57a	2271.6 ± 1275.06c	284.66 ± 6.44d	4842.21 ± 325.76b	5642.78 ± 158.75ab	4773.94 ± 1319.91b
42	Ethanol-D	64-17-5	C2H6O	968.1	197.711	1.04335	2232.42 ± 195.3ab	2631.56 ± 23.33a	2124.78 ± 315.26b	2234.27 ± 248.09ab	729.3 ± 7.01c	2458.26 ± 57.97ab	2379.13 ± 49.67ab	2695.73 ± 235.55a
43	1-Propanol-M	71-23-8	C3H8O	1055.7	256.868	1.26308	97.16 ± 0.71d	2517.43 ± 81.4b	3934.66 ± 254.74a	1001.12 ± 1364.01cd	180.57 ± 6.51d	2660.51 ± 68.96b	2464.3 ± 68.57b	1723.03 ± 233.16bc
44	1-Propanol-D	71-23-8	C3H8O	1055.7	256.868	1.11421	274.95 ± 4.5c	2610.72 ± 21.76a	2534.86 ± 176.62a	1487.09 ± 987.96b	356.7 ± 30.31c	2639.31 ± 71a	2580.01 ± 23.96a	2447.8 ± 32.67a
45	Butanol-M	71-36-3	C4H10O	1155.9	362.357	1.18159	191.15 ± 4.59d	389.62 ± 3.18c	564.08 ± 46.74a	367.09 ± 69.99c	247.47 ± 5.67d	376.92 ± 8.72c	406.15 ± 12.5c	482.56 ± 2.39b
46	Butanol-D	71-36-3	C4H10O	1153.2	358.917	1.38121	5.24 ± 0.24e	19.14 ± 0.66cd	47.66 ± 9.03a	19.63 ± 8.4cd	8.3 ± 1.23de	19.98 ± 1.48cd	24.07 ± 2.94bc	32.65 ± 0.61b
47	2-Methyl-1-propanol-M	78-83-1	C4H10O	1107.9	305.609	1.17258	150.89 ± 37.25f	771.68 ± 42.85cd	1191.93 ± 56.86b	583.32 ± 176.99d	376 ± 73.46e	859.66 ± 22.72c	943.04 ± 32.27c	2367.68 ± 115.88a
48	2-Methyl-1-propanol-D	78-83-1	C4H10O	1107.9	305.609	1.37214	58.31 ± 4.64b	141.1 ± 13.65b	282.71 ± 41.1b	97.62 ± 34.13b	169.46 ± 4.7b	151.38 ± 2.7b	180.99 ± 15.88b	1630.08 ± 239.26a
49	Tert-butanol-M	75-65-0	C4H10O	921.3	176.446	1.16221	422.14 ± 1.07b	420.3 ± 4.17b	479.83 ± 26.42a	429.05 ± 41.44b	196.51 ± 2.97c	422.36 ± 15.4b	424.89 ± 4.39b	413.02 ± 4.06b
50	Tert-butanol-D	75-65-0	C4H10O	916	174.212	1.3261	586.01 ± 7.41c	763.59 ± 14.03b	458.57 ± 69.6d	637.95 ± 37.59c	1078.21 ± 3.19a	825.74 ± 30.94b	807.59 ± 17.12b	819.69 ± 50.36b
51	1-Pentanol-M	71-41-0	C5H12O	1263.4	526.066	1.25915	556.4 ± 10.92e	1516.23 ± 30.44bc	1901.41 ± 6.29a	1761.91 ± 122.22a	940.17 ± 93.11d	1417.22 ± 50.03c	1454.98 ± 12.78c	1615.75 ± 64.57b
52	1-Pentanol-D	71-41-0	C5H12O	1264.2	527.393	1.25915	69 ± 5.81d	565.2 ± 15.07c	909.92 ± 62.97a	715.53 ± 100.84b	77.77 ± 27.65d	492.17 ± 42c	525.54 ± 20.81c	584.1 ± 25.63c
	Acids													
53	Acetic acid-M	64-19-7	C2H4O2	1462.7	882.662	1.05707	3575.76 ± 77.65cd	4157.19 ± 151.85bc	3251.41 ± 327.56de	2764.75 ± 154.37e	2600.67 ± 79.4e	4552.19 ± 548.98b	5739.65 ± 271.75a	4746.25 ± 253b
54	Acetic acid-D	64-19-7	C2H4O2	1461.9	880.951	1.16202	198.34 ± 3.06de	297.98 ± 38.17cd	200 ± 41.66de	140.11 ± 20.78e	103.12 ± 4.9e	333.24 ± 82.58bc	582.15 ± 62.74a	438.77 ± 56.4b
55	Propanoic acid-M	1979/9/4	C3H6O2	1536.9	1058.96	1.1159	149.61 ± 1.57e	1216.9 ± 164.37c	642.28 ± 99.67d	406.33 ± 79.59de	271.41 ± 16.48e	1686.1 ± 290.54b	2002.19 ± 61.48a	1331.75 ± 80.78c
56	Propanoic acid-D	1979/9/4	C3H6O2	1539.1	1064.666	1.26697	35.11 ± 3.37c	86.51 ± 21.87b	39.57 ± 9.12c	34.8 ± 1.68c	37.01 ± 2.92c	118.52 ± 33.45b	166.87 ± 14.17a	79.73 ± 15.73b
	Furans													
57	2-Methyl-3-sulfanylfuran	28588-74-1	C5H6OS	1251.8	505.503	1.14627	777.24 ± 12.07a	201.4 ± 7.44c	128.62 ± 5.97cd	146.17 ± 38.89cd	551.07 ± 80.78b	158.96 ± 4.29cd	150.45 ± 4.75cd	87.16 ± 4.5d
58	2-Pentylfuran	3777-69-3	C9H14O	1223.3	458.485	1.24133	393.9 ± 176.76c	2592.77 ± 21.61a	2764.49 ± 63.98a	2531.56 ± 655.4a	1201.03 ± 635.72b	2914.43 ± 33.21a	2924.14 ± 41.21a	3410.22 ± 168.52a
	others													
59	Acrylonitrile-M	107-13-1	C3H3N	1021.9	229.979	1.04815	1278.5 ± 64.66cd	1800.39 ± 125.23bc	2722.69 ± 495.63a	1360.8 ± 377.12cd	641.87 ± 20.69d	1874.34 ± 103.7bc	2263.9 ± 107.44ab	2298.48 ± 590.32ab
60	Acrylonitrile-D	107-13-1	C3H3N	1027	233.813	1.0919	92.26 ± 11.71bc	139.08 ± 8.5ab	157.97 ± 42.51a	75.17 ± 26.04c	33.21 ± 1.4c	160.81 ± 11.03a	192.43 ± 10.41a	137.82 ± 43.76ab
61	Alpha-Pinene	80-56-8	C10H16	1001.4	215.038	1.29827	392.4 ± 16.46d	1081.36 ± 50.79c	1388.18 ± 67.87b	450.42 ± 97.04d	179.43 ± 9.66d	1374.26 ± 96.85b	1700.53 ± 63.38a	938.25 ± 260.51c
62	1	unidentified		920.3	268.881	1.27507	577.12 ± 35.7a	265.53 ± 36.84b	76.03 ± 55.05c	515.83 ± 27.69a	314.4 ± 1.34b	245.14 ± 33.71b	155.31 ± 14.62bc	294.9 ± 157.49b
63	2	unidentified		846.3	236.999	1.46736	717.22 ± 6.54c	587.82 ± 29.74d	618.87 ± 95.37d	772.89 ± 43.51bc	913.87 ± 8.57a	528.73 ± 11.45d	599.98 ± 6.39d	829.46 ± 37.64ab
64	3	unidentified		902.5	260.868	1.28513	322.69 ± 9.51b	227.98 ± 6.64c	162.8 ± 31.41d	325.65 ± 56.9b	535.89 ± 8.79a	280.89 ± 5.1bc	306.35 ± 29.71b	280.04 ± 18.37bc

Notes: RI, retention indices calculated in the experiment; Rt, retention time (s); Dt, drift time (ms). -M stands for monomers of flavor compounds; -D stands for dimer of flavor compounds. 1, 2, 3 are unidentified volatile compounds. Different letters in the figure indicate statistically significant differences among groups (*p* < 0.05).

**Table 2:** The glycated peptides from OP identified by nano-LC/HRMS.

NO.	Peptide Sequence	Length	*m*/*z*	Charge	MH + (Da)	M_Theo (Da)	ΔM (ppm)	Mass Shift	RT	Accession Protein Number	Accession Protein Description
CON											
1	KAFGHENEALVR**K**	13	554.2937	3	1660.8666	1660.8654	0.72	162.0654	14.48	A0A8W8MFC2;K1PY28	EF-hand domain-containing protein; Sarcoplasmic calcium-binding protein
2	DVIDTN**K**DRTIDE	13	565.9363	3	1695.7943	1695.7920	1.36	162.0542	22.49	A0A8W8MFC2;K1PY28	EF-hand domain-containing protein; Sarcoplasmic calcium-binding protein
3	DSRAATSPGELGVTIEGP**K**E	20	726.0271	3	2176.0668	2176.0617	2.34	162.0558	35.10	A0A8W8LXP5	Filamin-C
4	NLHELVGD**K**AKGVQVNF	17	508.2697	4	2030.0569	2030.0554	0.76	162.0519	37.52	A0A8W8MFC2;K1PY28	EF-hand domain-containing protein; Sarcoplasmic calcium-binding protein
110-1											
1	LQ**K**EKSCTIK	10	466.2490	3	1396.7324	1396.7352	−2.01	162.1239	15.85	A0A8W8HUR0	AAA+ ATPase domain-containing protein
2	D**K**DGKGKIPEEY	12	514.2510	3	1540.7386	1540.7378	0.52	162.1039	17.47	A0A8W8INF3	EF-hand domain-containing protein
3	DSRAATSPGELGVTIEGP**K**E	20	726.0264	3	2176.0646	2176.0617	1.33	162.0503	34.80	A0A8W8LXP5	Filamin-C
4	AF**K**AFGHENEALVRK	15	470.4984	4	1878.9717	1878.9709	0.43	162.0519	23.81	A0A8W8MFC2;K1PY28	EF-hand domain-containing protein; Sarcoplasmic calcium-binding protein
5	SPFKVEVGPA**K**T	12	474.5892	3	1421.7530	1421.7523	0.48	162.1388	29.49	K1PW06	Filamin-C
110-3											
1	GESGLPG**R**DGDSGPPGRQGGRG	22	565.7609	4	2260.0216	2260.0186	1.35	162.0546	11.50	A0A8W8NCS6	Collagen alpha-2(I) chain
2	SRN**K**FTNLH	9	426.8860	3	1278.6434	1278.6437	−0.23	162.0522	13.54	A0A8W8MFC2;K1PY28	EF-hand domain-containing protein; Sarcoplasmic calcium-binding protein
3	D**K**DGKGKIPEEY	12	514.2509	3	1540.7382	1540.7377	0.29	162.0526	17.32	A0A8W8INF3	EF-hand domain-containing protein
4	D**K**GNKGTIPVED	12	478.9035	3	1434.6961	1434.6959	0.11	162.0523	18.58	A0A8W8P6I7	EF-hand domain-containing protein
5	A**K**IETKQNPDGTVGVT	16	607.3149	3	1819.9301	1819.9284	0.92	162.0509	19.30	A0A8W8LXP5;K1PW06	Filamin-C; Filamin-C
6	DVIDTN**K**DRTIDE	13	565.9360	3	1695.7934	1695.7920	0.82	162.0533	22.27	A0A8W8MFC2;K1PY28	EF-hand domain-containing protein; Sarcoplasmic calcium-binding protein
7	AF**K**AFGHENEALVRK	15	470.4981	4	1878.9704	1878.9709	−0.29	162.0534	23.57	A0A8W8MFC2;K1PY28	EF-hand domain-containing protein; Sarcoplasmic calcium-binding protein
8	SPF**K**VEVGPAKT	12	474.5889	3	1421.7522	1421.7523	−0.04	162.0526	29.36	K1PW06	Filamin-C
9	DIVSEWV**K**FVTEEDTGKK	18	572.7855	4	2288.1203	2288.1181	0.95	162.0517	64.45	A0A8W8MFC2	EF-hand domain-containing protein
10	YPM**K**IVS**R**L	9	723.8756	2	1446.7438	1446.7396	2.90	324.1090	75.61	A0A8W8KEF5	Cation-transporting P-type ATPase N-terminal domain-containing protein
110-5											
1	**R**RGESGPNGEPGRTGPPGPRGPRG	24	519.2585	5	2592.2636	2592.26230	0.51	162.0527	9.68	K1PDS7	Collagen alpha-2(I) chain
2	G**K**DGPAGEHGSPGPLGPR	18	466.7268	4	1863.8852	1863.88323	1.03	162.0538	14.79	K1PT11	Collagen alpha-2(I) chain
3	G**R**PGEEGQPGAPGHQGPLGPR	21	562.0208	4	2245.0612	2245.05932	0.83	162.0526	15.66	K1PDS7	Collagen alpha-2(I) chain
4	VTVEGPSKV**K**L	11	440.2539	3	1318.7471	1318.74646	0.46	162.0527	28.46	K1PW06	Filamin-C
5	SIDLSKV**K**VV	10	417.2465	3	1249.7250	1249.72500	0.01	162.0541	38.28	A0A8W8LXP5;K1PW06	Filamin-C; Filamin-C
6	DIVSEWV**K**FVTEEDSSKK	18	572.7855	4	2288.1203	2288.11809	0.95	162.0521	64.54	K1PY28	Sarcoplasmic calcium-binding protein
130-1											
1	**R**GESGPNGEPGRTGPPGPRGPRG	23	609.7960	4	2436.1620	2436.1612	0.34	162.0538	10.62	K1PDS7	Collagen alpha-2(I) chain
2	KAFGHENEALVR**K**	13	554.2939	3	1660.8671	1660.8654	1.05	162.0659	14.51	A0A8W8MFC2;K1PY28	EF-hand domain-containing protein; Sarcoplasmic calcium-binding protein
3	G**R**PGEEGQPGAPGHQGPLGPR	21	562.0209	4	2245.0619	2245.0593	1.16	162.0494	15.60	K1PDS7	Collagen alpha-2(I) chain
4	DKDG**K**G**K**IPEEY	12	568.2693	3	1702.7933	1702.7906	1.62	324.1212	17.25	A0A8W8INF3	EF-hand domain-containing protein
5	D**K**GNKGTIPVED	12	478.9038	3	1434.6968	1434.6959	0.62	162.0533	18.69	A0A8W8P6I7	EF-hand domain-containing protein
6	DSRAATSPGELGVTIEGP**K**E	20	726.0261	3	2176.0636	2176.0616	0.91	162.0503	34.87	A0A8W8LXP5	Filamin-C
7	DIVSEWV**K**FVTEEDSSKK	18	572.7858	4	2288.1212	2288.1181	1.37	162.0531	64.56	K1PY28	Sarcoplasmic calcium-binding protein
130-3											
1	**R**GESGPNGEPGRTGPPGPRGPRG	23	609.7965	4	2436.1642	2436.1612	1.25	162.0532	10.63	K1PDS7	Collagen alpha-2(I) chain
2	SRN**K**FTNLH	9	426.8861	3	1278.6439	1278.6437	0.13	162.0523	13.16	A0A8W8MFC2;K1PY28	EF-hand domain-containing protein; Sarcoplasmic calcium-binding protein
3	G**R**PGEEGQPGAPGHQGPLGPR	21	562.0208	4	2245.0614	2245.0593	0.94	162.0520	15.44	K1PDS7	Collagen alpha-2(I) chain
4	AIRAGYDINK**K**A	12	494.6036	3	1481.7963	1481.7959	0.27	162.0528	16.74	A0A8W8N9M9	Natterin-3
5	D**K**DGKGKIPEEY	12	514.2510	3	1540.7386	1540.7378	0.52	162.0526	17.25	A0A8W8INF3	EF-hand domain-containing protein
6	A**K**IETKQNPDGTVGVT	16	607.3152	3	1819.9310	1819.9284	1.42	162.0534	19.18	A0A8W8LXP5;K1PW06	Filamin-C; Filamin-C
7	DVIDTN**K**DRTIDE	13	565.9360	3	1695.7936	1695.7920	0.93	162.0592	22.23	A0A8W8MFC2;K1PY28	EF-hand domain-containing protein; Sarcoplasmic calcium-binding protein
8	VTVEGPSKV**K**L	11	440.2536	3	1318.7463	1318.7465	−0.09	162.0520	28.24	K1PW06	Filamin-C
9	SPF**K**VEVGPAKT	12	474.5889	3	1421.7521	1421.7523	−0.1	162.0534	29.41	K1PW06	Filamin-C
10	DSRAATSPGELGVTIEGP**K**E	20	726.0267	3	2176.0657	2176.0616	1.84	162.0502	34.71	A0A8W8LXP5	Filamin-C
130-5											
1	RLPG**K**KKPR	9	419.9263	3	1257.7644	1257.7638	0.52	162.0526	7.86	A0A8W8IGL5	Uncharacterized protein
2	GQ**R**GIPGERGRDGDRGSNG	19	530.5034	4	2118.9918	2118.9872	2.19	162.0441	7.91	K1PDS7	Collagen alpha-2(I) chain
3	APPVEEGGGK**K**	11	410.8789	3	1230.6223	1230.6213	0.80	162.0538	8.27	A0A8W8KP76	Myosin heavy chain, striated muscle
4	**R**RGESGPNGEPGRTGPPGPRGPRG	24	519.2582	5	2592.2618	2592.2623	−0.19	162.0499	9.24	K1PDS7	Collagen alpha-2(I) chain
5	GESGLPG**R**DGDSGPPGRQGGRG	22	565.7606	4	2260.0207	2260.0186	0.92	162.0521	11.00	A0A8W8NCS6	Collagen alpha-2(I) chain
6	SRN**K**FTNLH	9	426.8861	3	1278.6438	1278.6437	0.06	162.0525	13.20	A0A8W8MFC2;K1PY28	EF-hand domain-containing protein; Sarcoplasmic calcium-binding protein
7	G**R**PGEEGQPGAPGHQGPLGPR	21	566.0197	4	2261.0568	2261.0542	1.13	162.0534	13.93	K1PDS7	Collagen alpha-2(I) chain
8	G**K**DGPAGEHGSPGPLGPR	18	466.7266	4	1863.8847	1863.8832	0.77	162.0541	14.70	K1PT11	Collagen alpha-2(I) chain
9	YISLEELYKIMTT**K**	14	474.2512	4	1893.9828	1893.9766	3.28	162.0582	15.87	A0A8W8JIB3	EF-hand domain-containing protein
10	SLYNKENKHVPL**K**	13	433.7378	4	1731.9295	1731.9276	1.08	162.0565	16.00	A0A8W8MFC2	EF-hand domain-containing protein
11	D**K**DGKGKIPEEY	12	514.2512	3	1540.7389	1540.7378	0.76	162.0527	17.10	A0A8W8INF3	EF-hand domain-containing protein
12	NLHELVGD**K**AKGVQVNF	17	508.2692	4	2030.0548	2030.0554	−0.26	162.0427	36.99	A0A8W8MFC2;K1PY28	EF-hand domain-containing protein; Sarcoplasmic calcium-binding protein
13	DEFVYAF**K**AFGHEN	14	612.6097	3	1835.8145	1835.8123	1.19	162.0542	48.75	A0A8W8MFC2;K1PY28	EF-hand domain-containing protein; Sarcoplasmic calcium-binding protein
14	DIVSEWV**K**FVTEEDSS**K**K	18	613.2983	4	2450.1735	2450.1709	1.06	324.1299	63.71	K1PY28	Sarcoplasmic calcium-binding protein

Note: **K/R** indicates that the amino acid is modified by glycosylation.

## Data Availability

The original contributions presented in this study are included in the article and Supplementary Materials. Further inquiries can be directed to the corresponding authors.

## Supplementary Materials

The following supporting information can be downloaded at: https://www.mdpi.com/article/10.3390/foods15020236/s1, Figure S1. (A–D) Annotated MS/MS spectra of identified glycosylated peptides from the CON group. Figure S2. (A–E) Annotated MS/MS spectra of identified glycosylated peptides from the 110-1 group. Figure S3. (A–J) Annotated MS/MS spectra of identified glycosylated peptides from the 110-3 group. Figure S4. (A–F) Annotated MS/MS spectra of identified glycosylated peptides from the 110-5 group. Figure S5. (A–G) Annotated MS/MS spectra of identified glycosylated peptides from the 130-1 group. Figure S6. (A–J) Annotated MS/MS spectra of identified glycosylated peptides from the 130-3 group. Figure S7. (A–N) Annotated MS/MS spectra of identified glycosylated peptides from the 130-5 group.
