# Supplementary material for: Combination of GC-IMS and Nano-LC/HRMS Reveals the Mechanism of Superheated Steam Glycosylation Modification in Improving Oyster Peptide Flavor
Source: Foods. 2026 Jan 9;15(2):236. doi: 10.3390/foods15020236 (PMC12840250; doi:10.3390/foods15020236)
Supplement: Supplementary file 1 [file foods-15-00236-s001.zip › foods-3964532-supplementary.pdf]

CON:

(A) KAFGHENEALVR**K**

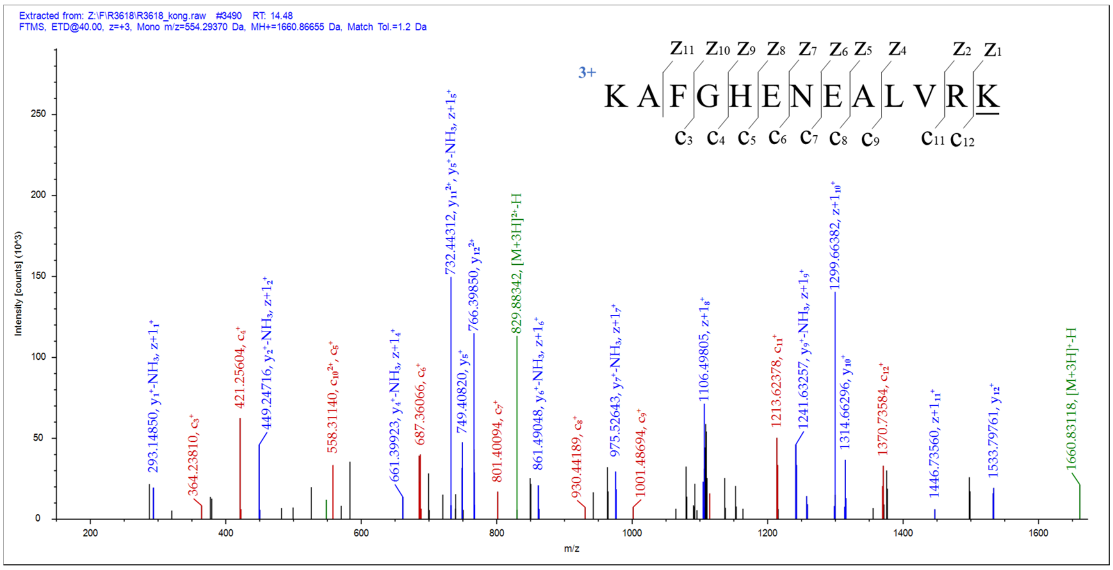

(B) DVIDTN**K**DRTIDE

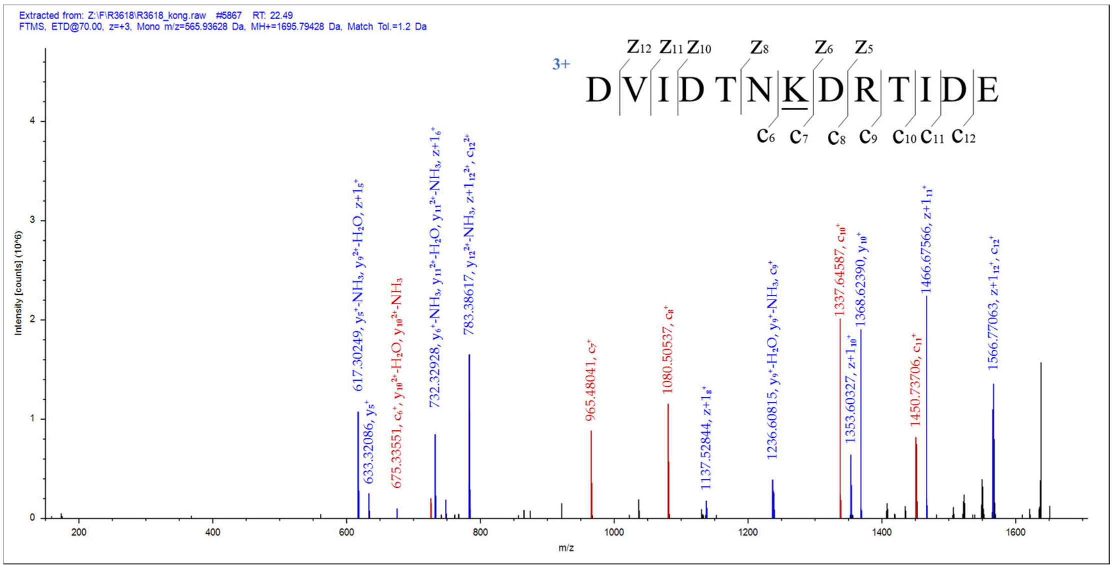

(C) DSRAATSPGELGVTIEGP**K**E

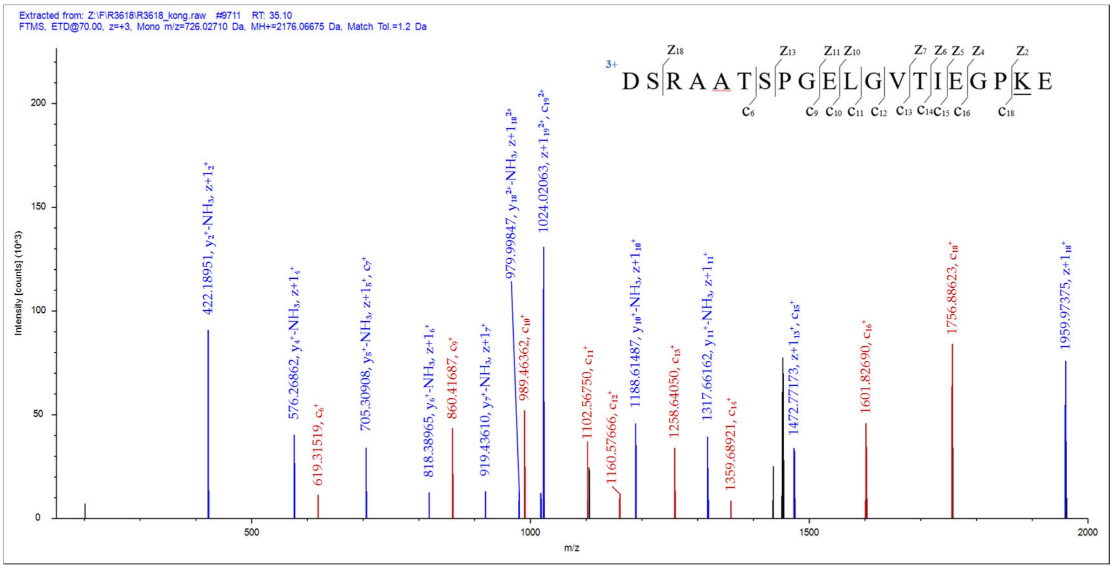

(D) NLHELVGD**K**AKGVQVNF

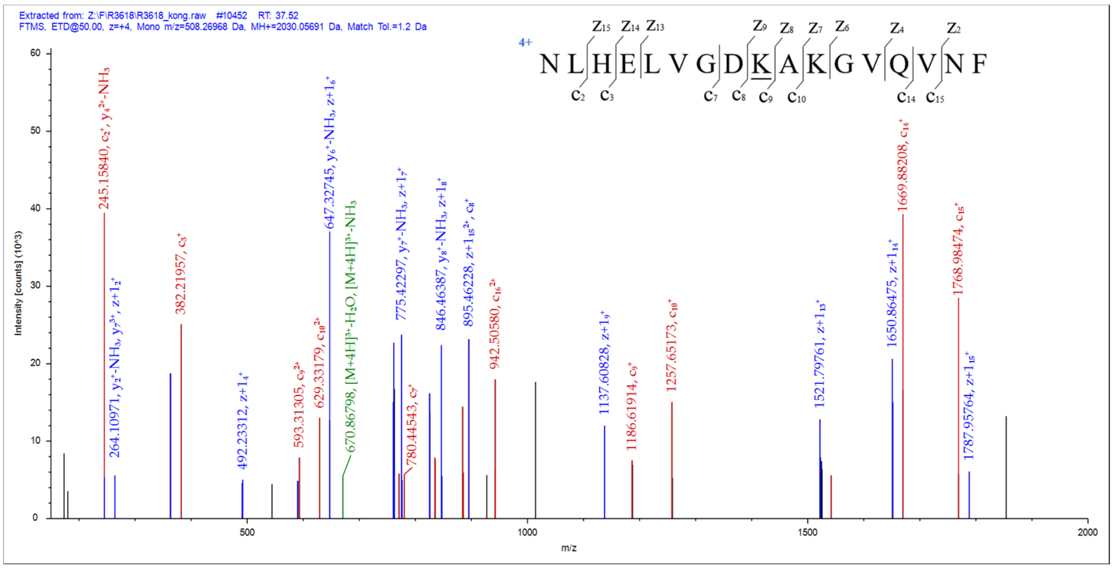

Fig. S1. (A-D) Annotated MS/MS spectra of identified glycosylated peptides from the CON group.

110-1：

(A) LQ**K**EKSCTIK

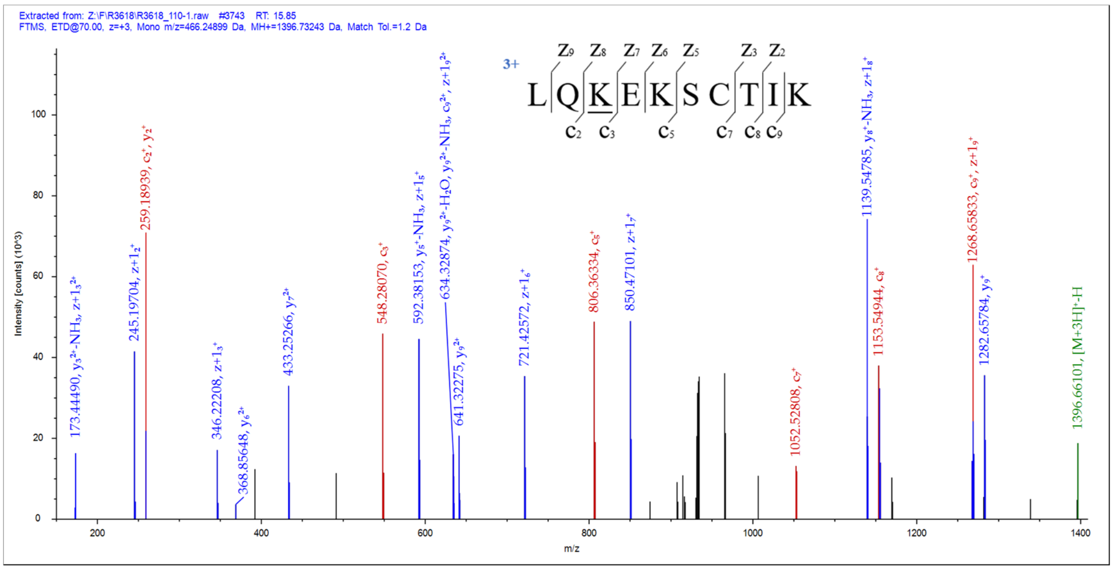

(B) D**K**DGKGKIPEEY

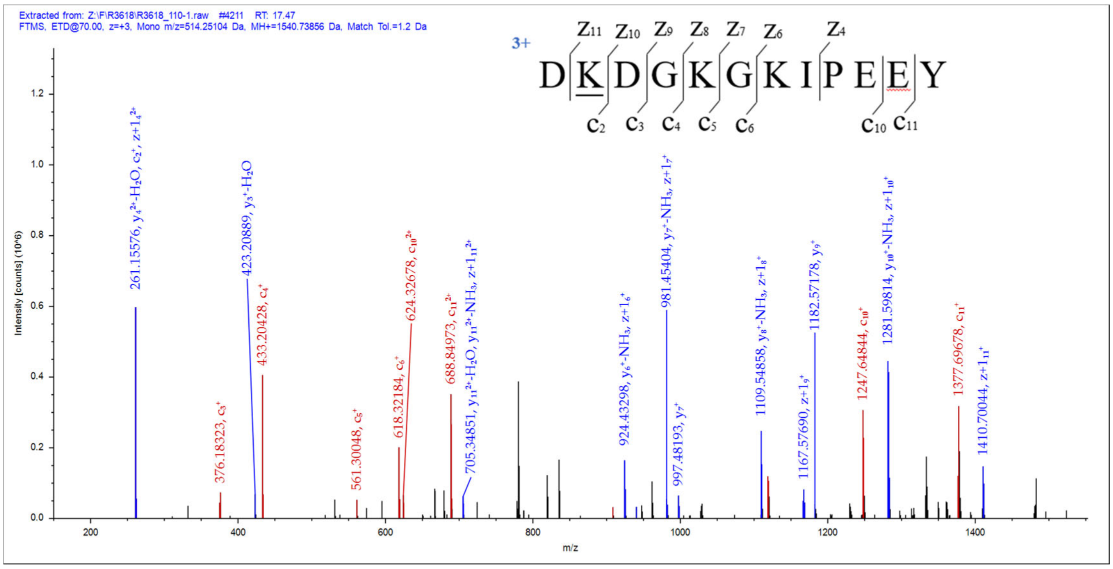

(C) DSRAATSPGELGVTIEGP**K**E

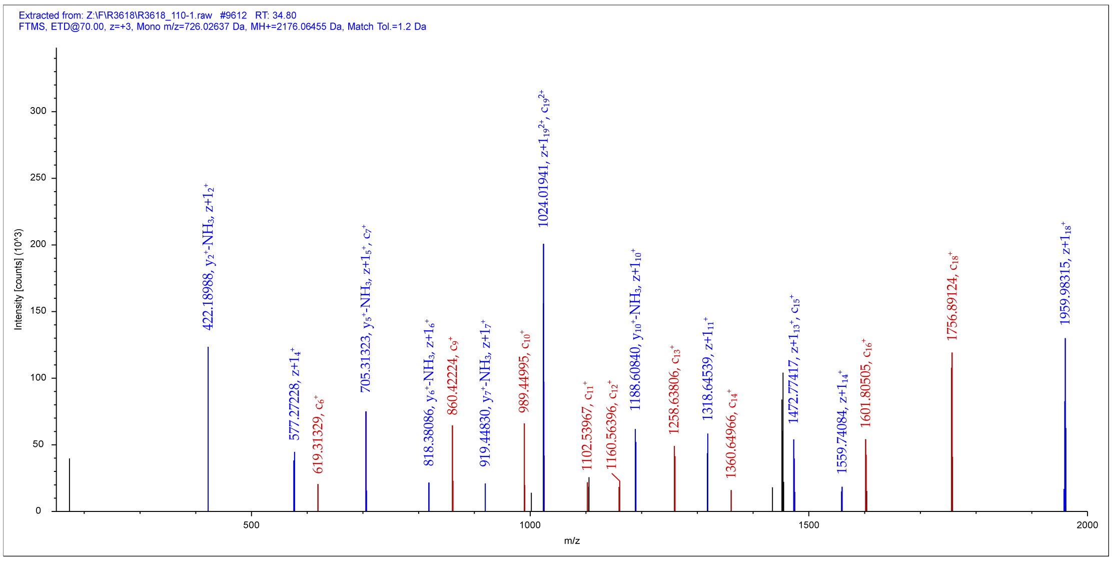

(D) AF**K**AFGHENEALVRK

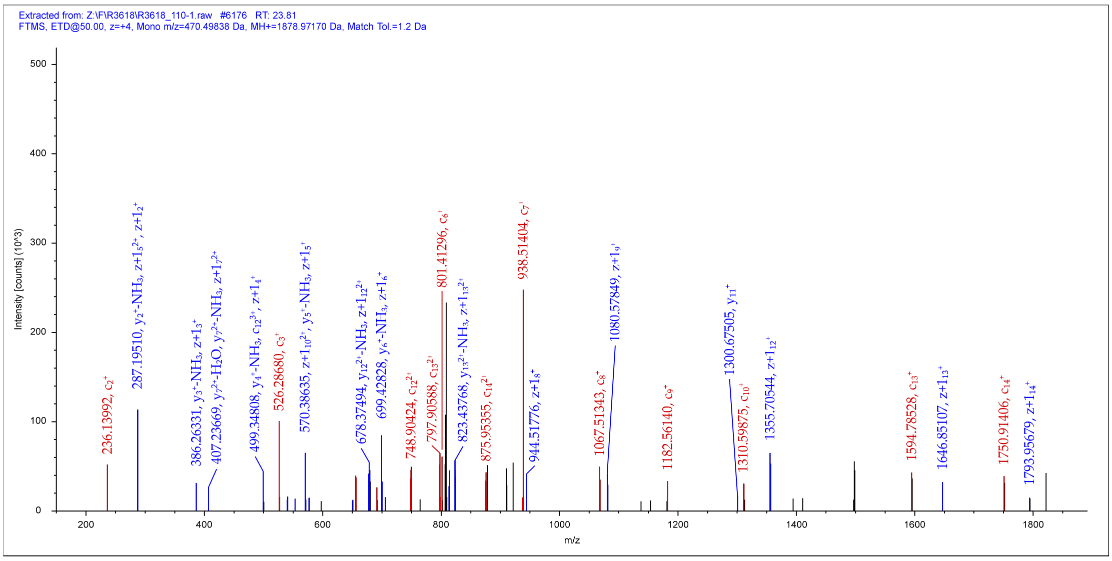

(E) SPFKVEVGPA**K**T

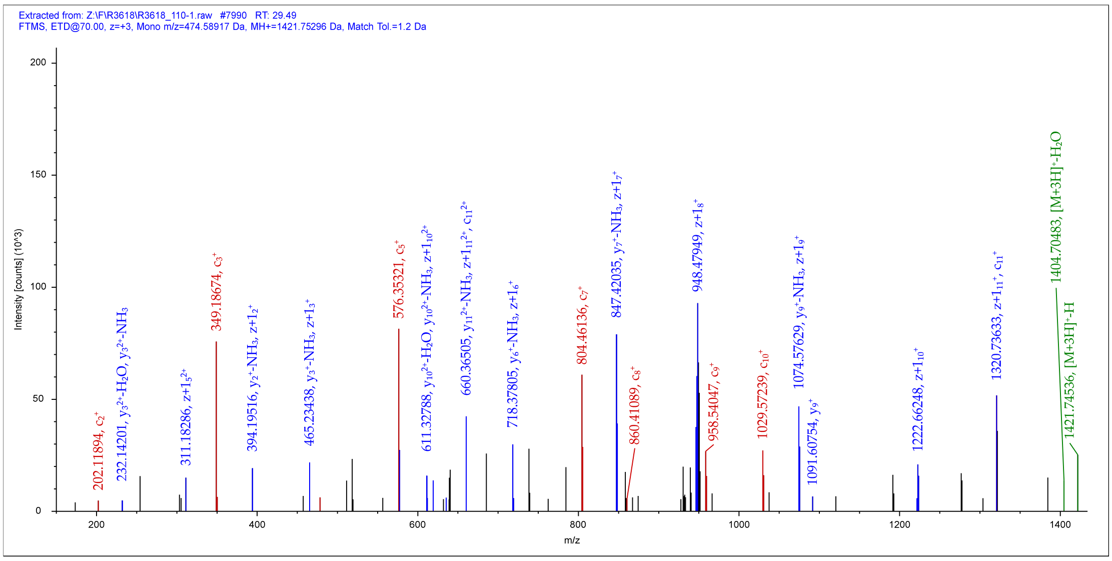

Fig. S2. (A-E) Annotated MS/MS spectra of identified glycosylated peptides from the 110-1 group.

110-3：

(A) GESGLPG**R**DGDSGPPGRQGGRG：

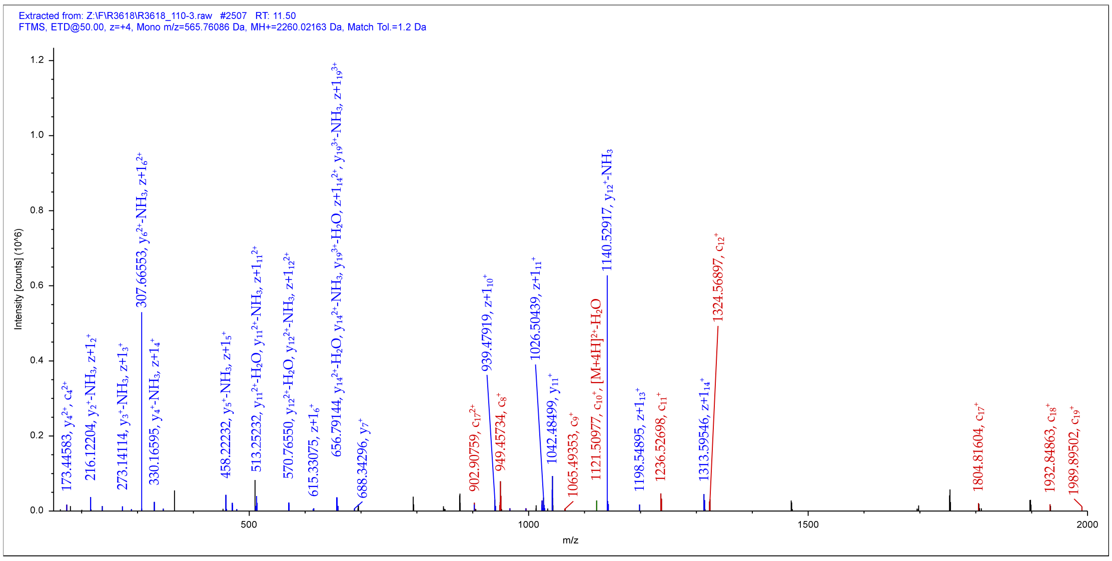

(B) SRN**K**FTNLH

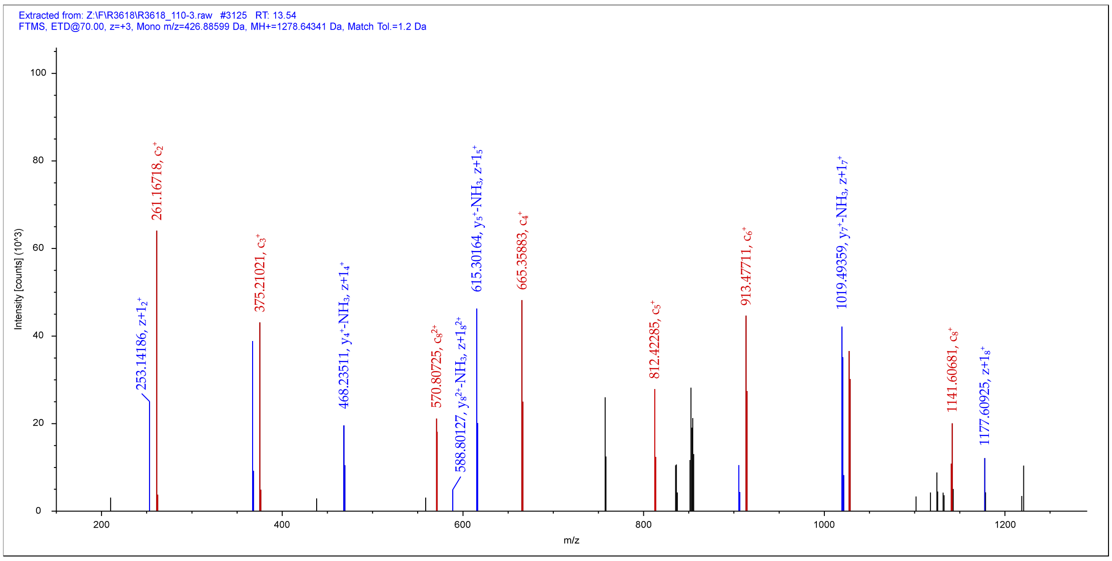

(C) D**K**DGKGKIPEEY

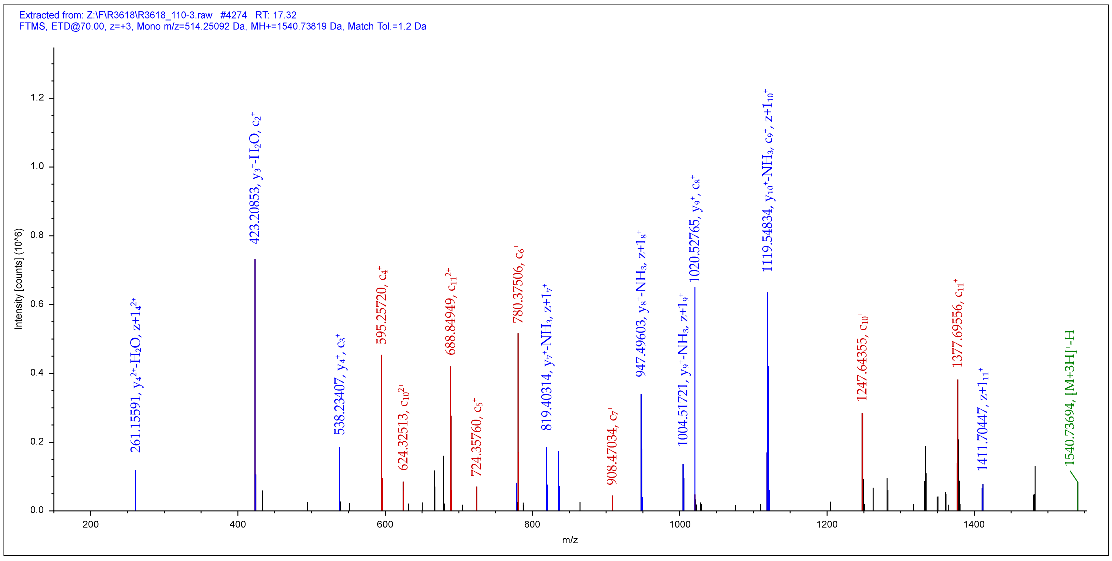

(D) D**K**GNKGTIPVED

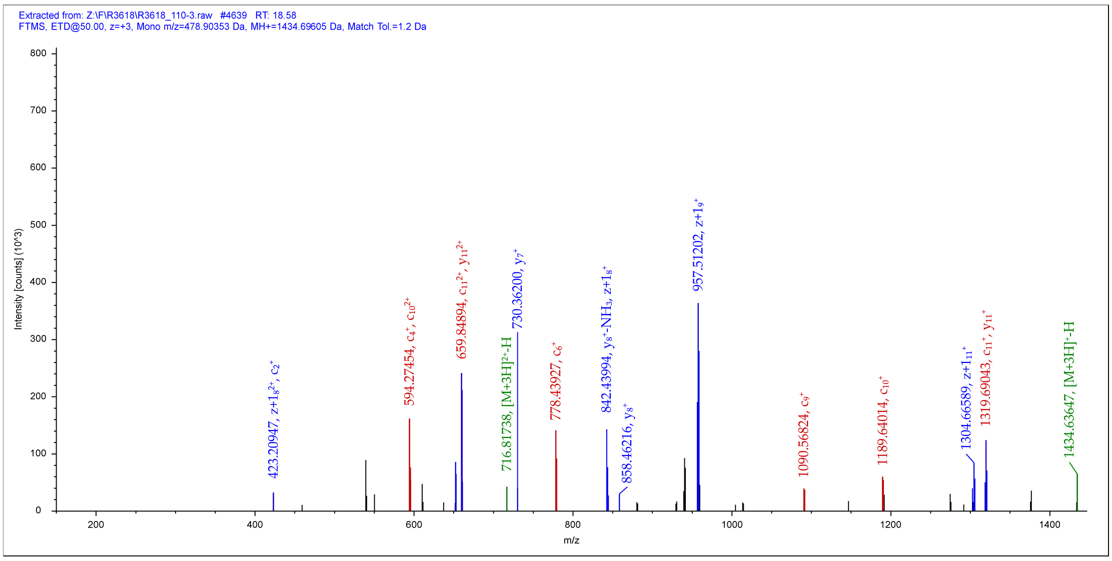

(E) A**K**IETKQNPDGTVGVT

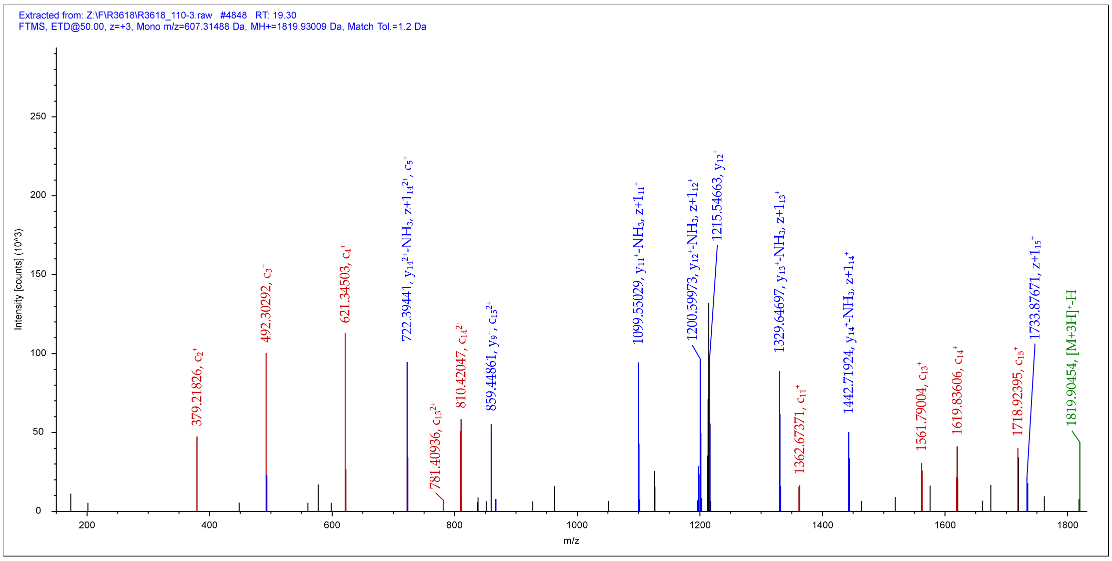

(F) DVIDTN**K**DRTIDE

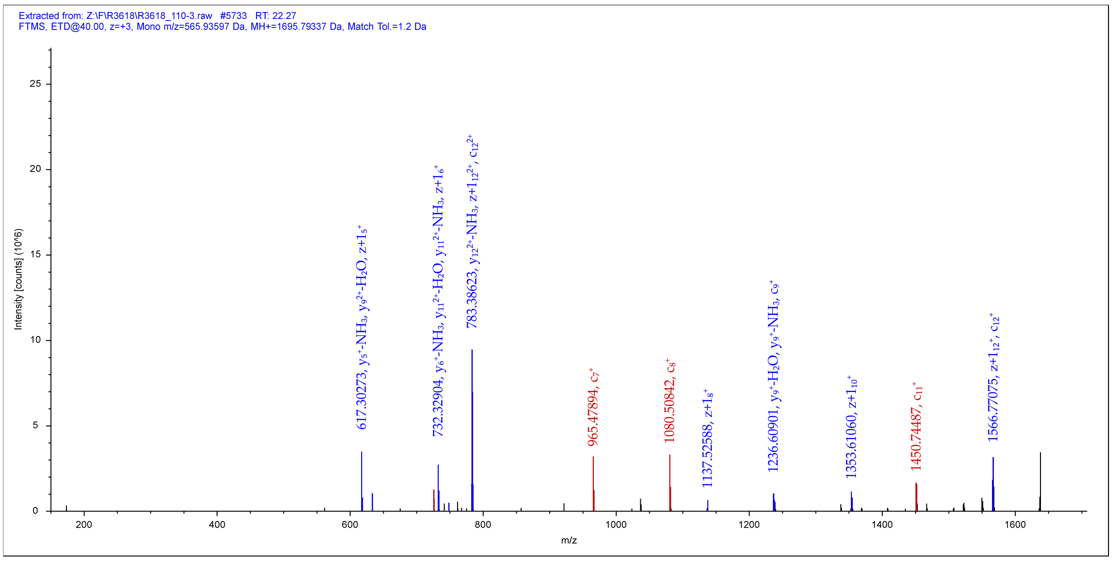

(G) AF**K**AFGHENEALVRK

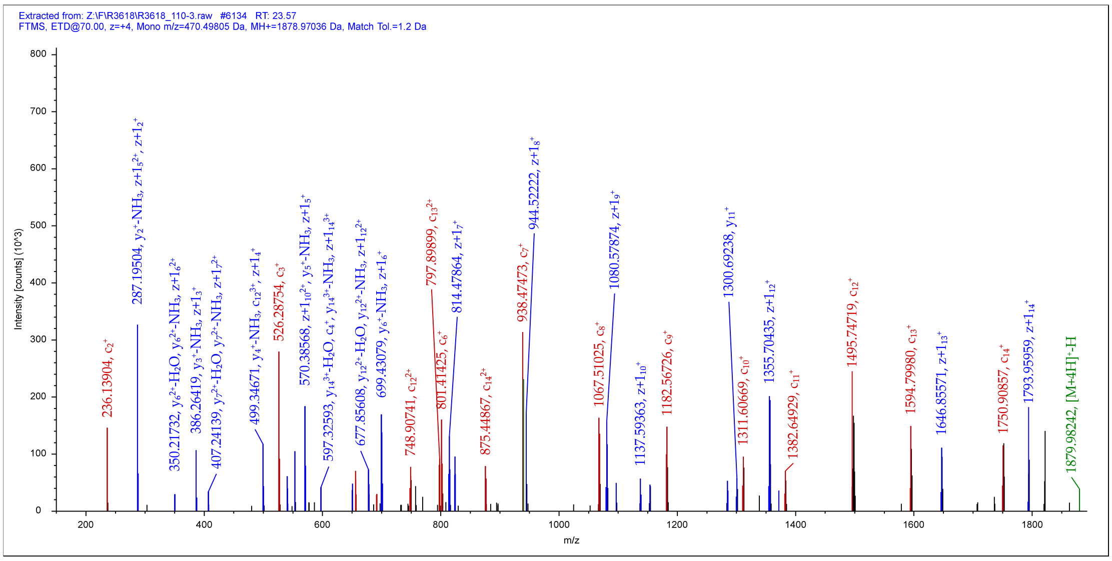

(H) SPF**K**VEVGPAKT

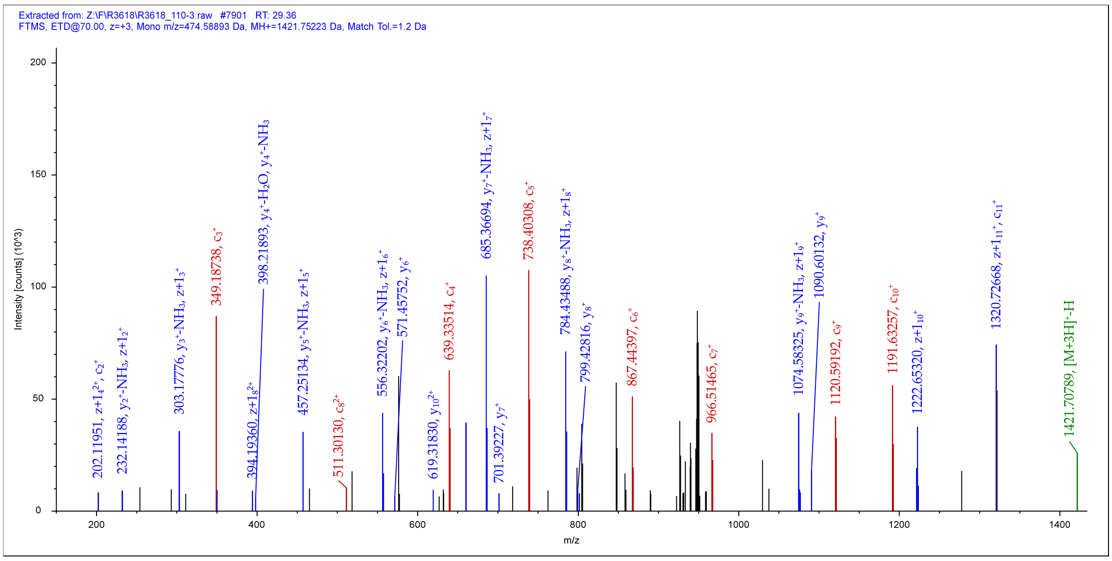

(I) DIVSEWVV**K**FVTEEDTGKK

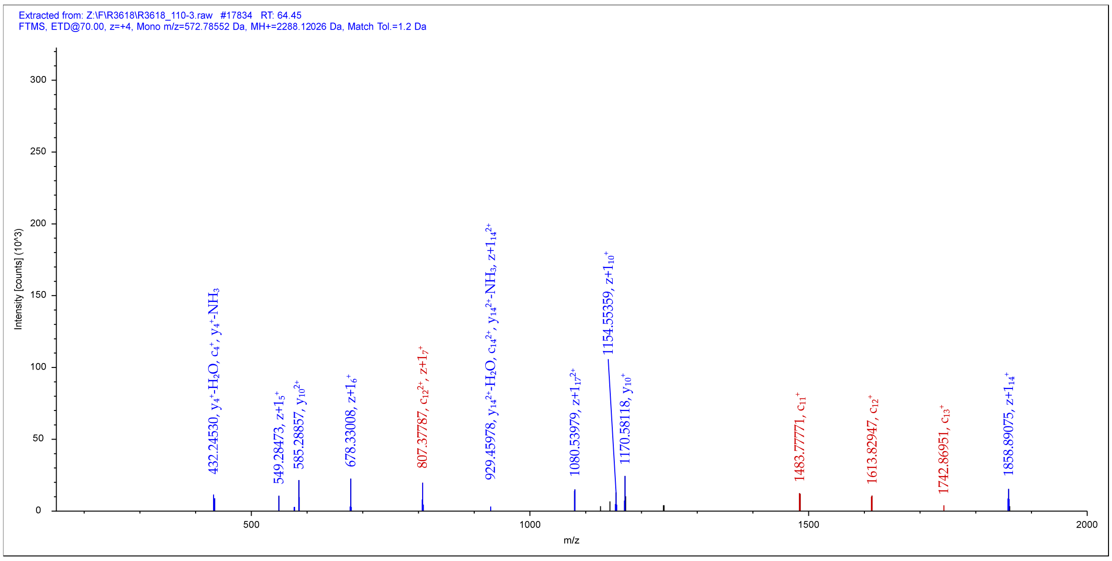

(J) YPM**K**IVS**R**L

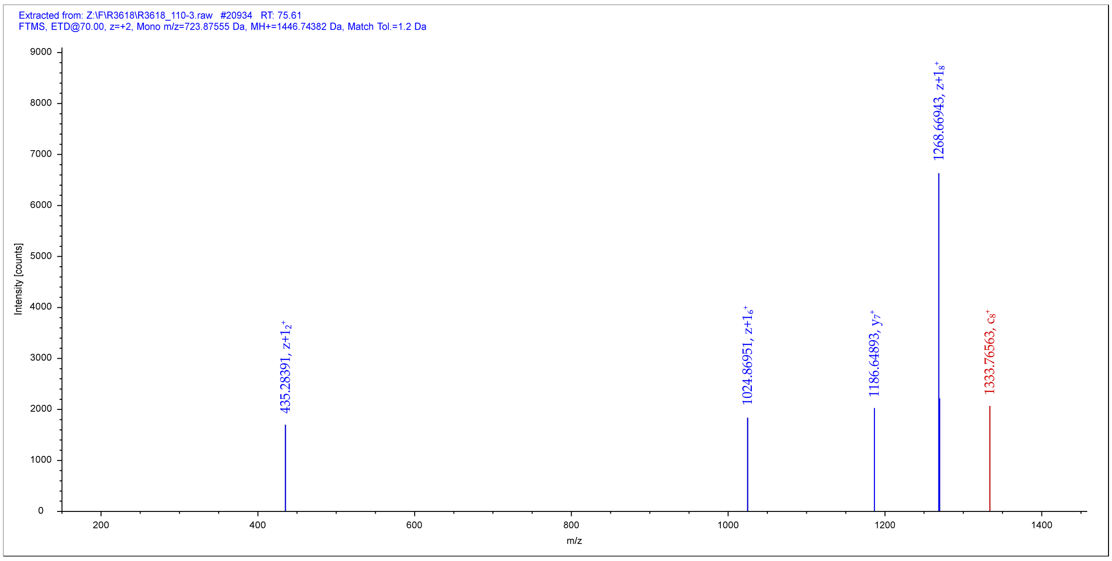

Fig. S3. (A-J) Annotated MS/MS spectra of identified glycosylated peptides from the 110-3 group.

110-5:

(A) RRGESGPNGEPGRTGPPGPRGPRG

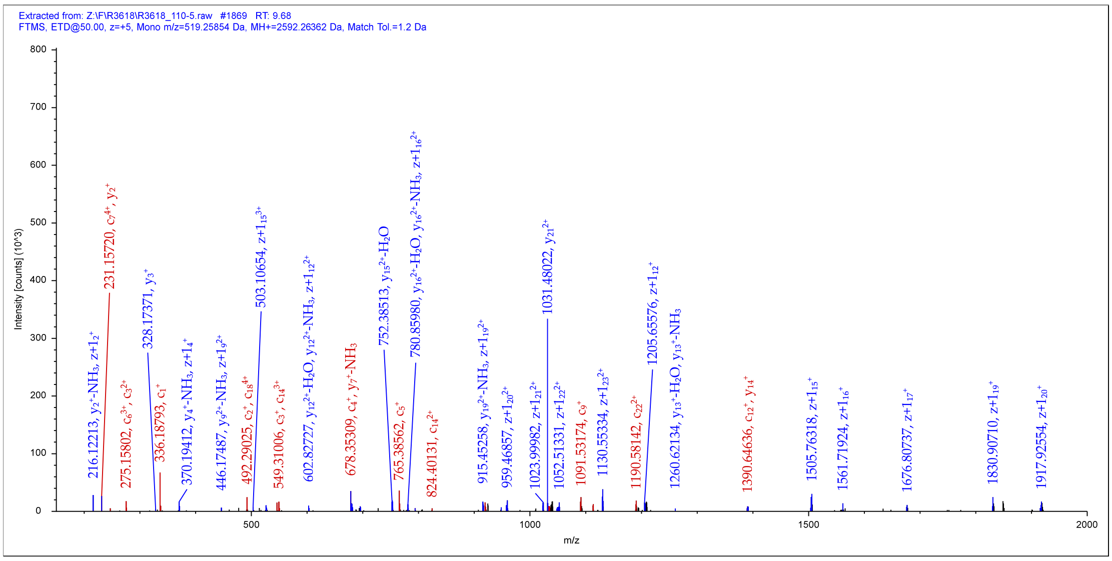

(B) GKDGPAGEHGSPGPLGPR

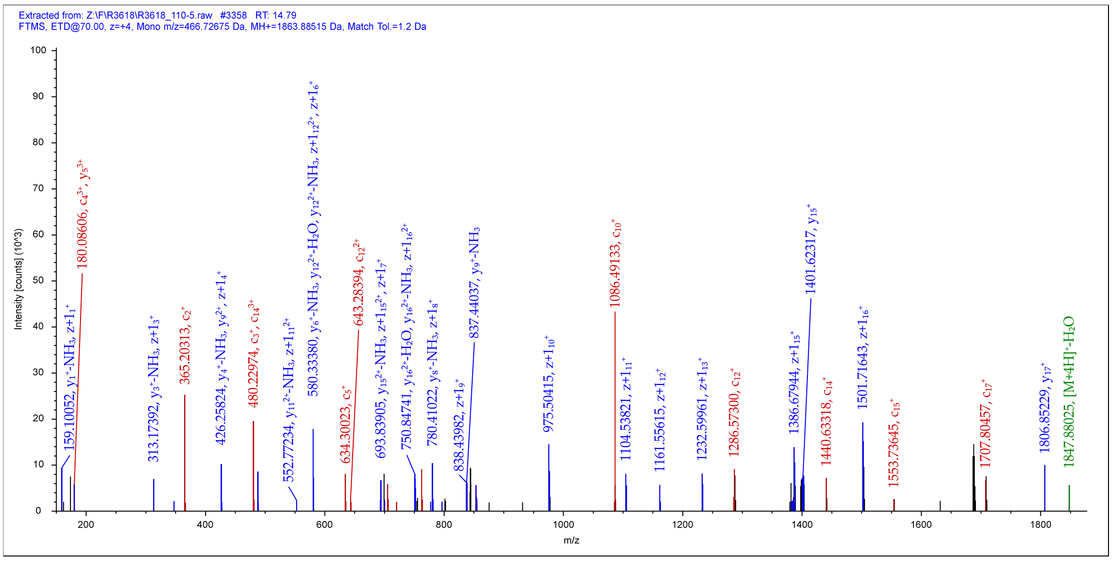

(C) GRPGEEGQPGAPGHQGPLGPR

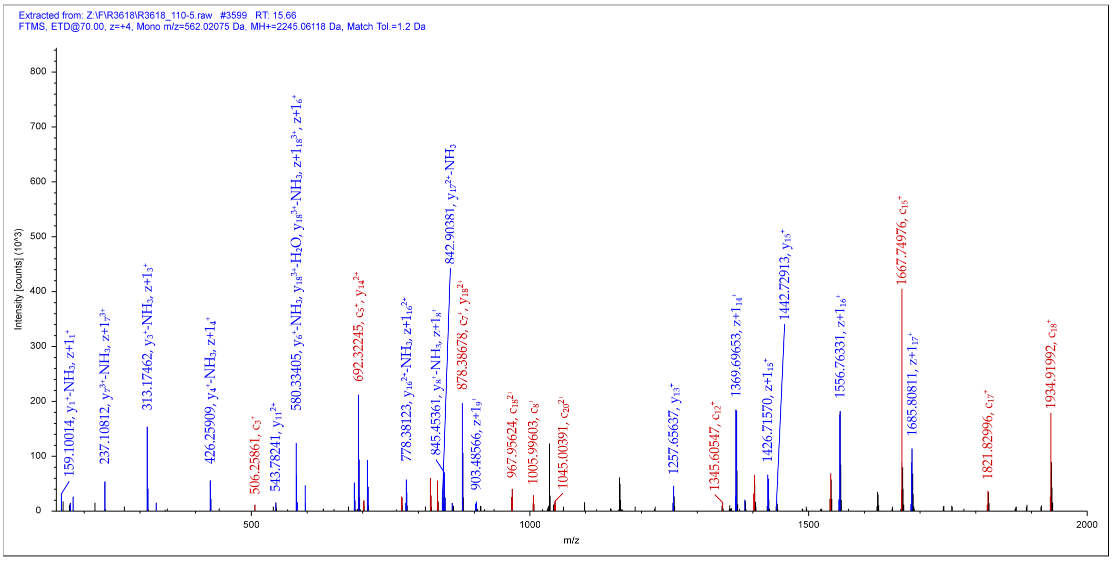

(D) VTVEGPSKV**K**L

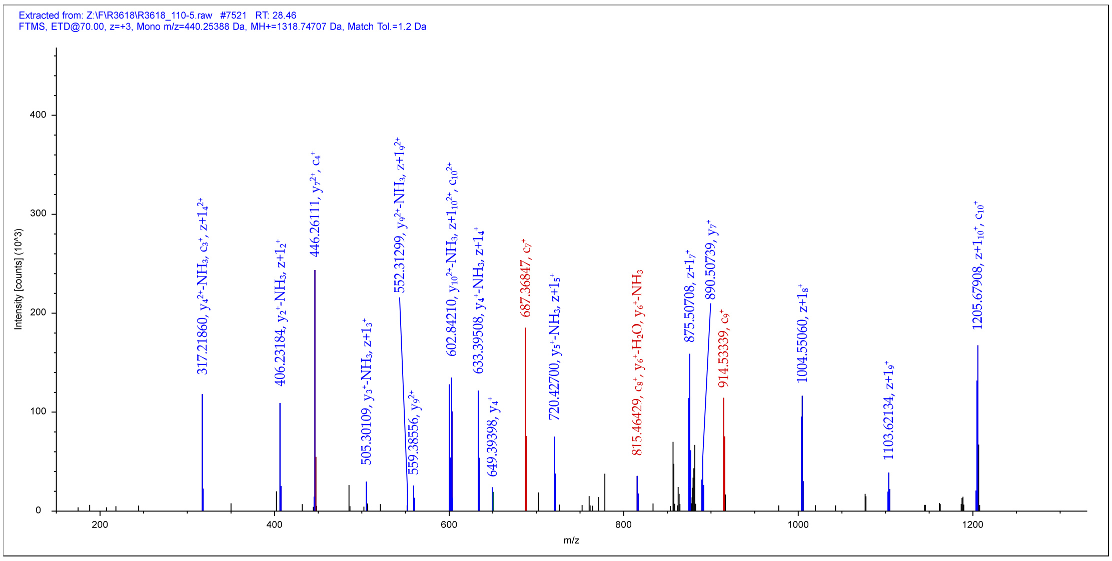

(E) SIDLSKV**K**VV

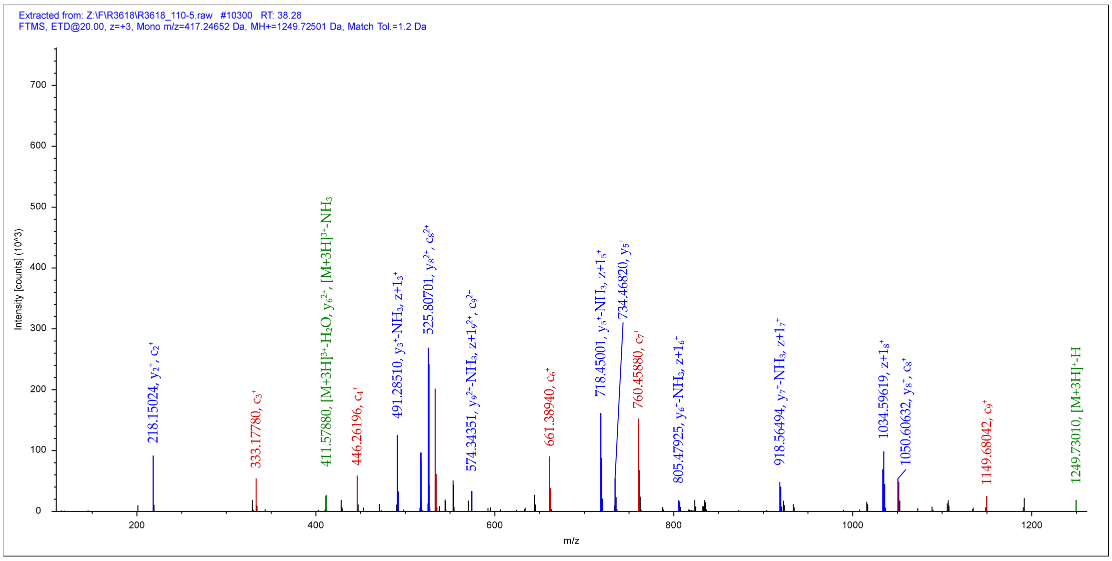

(F) DIVSEWV**K**FVTEEDSSKK

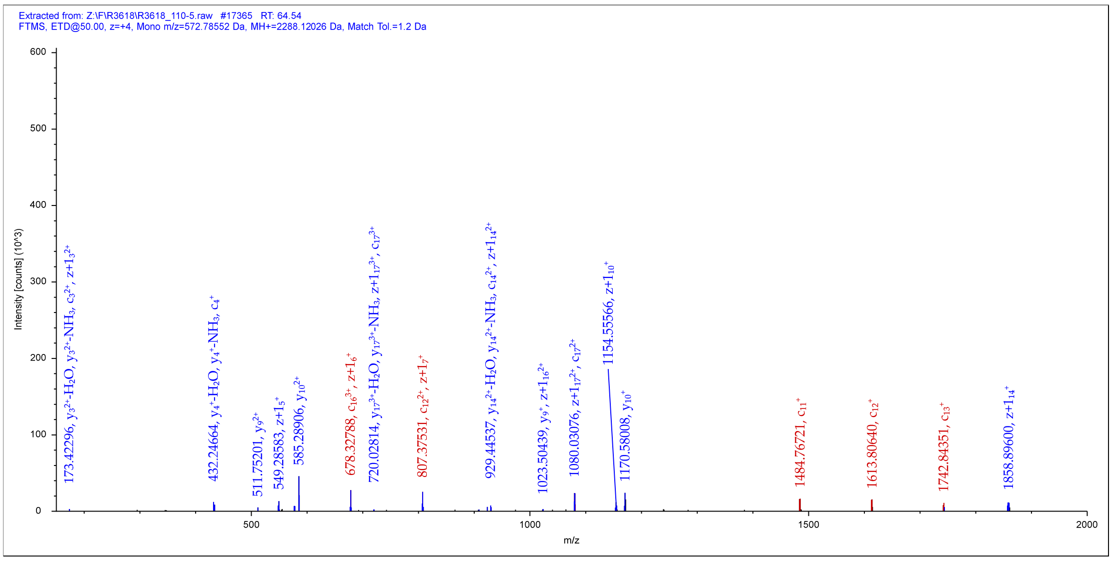

Fig. S4. (A-F) Annotated MS/MS spectra of identified glycosylated peptides from the 110-5 group.

130-1:

(A) **R**GESGPNGEPGRTGPPGPRGPRG

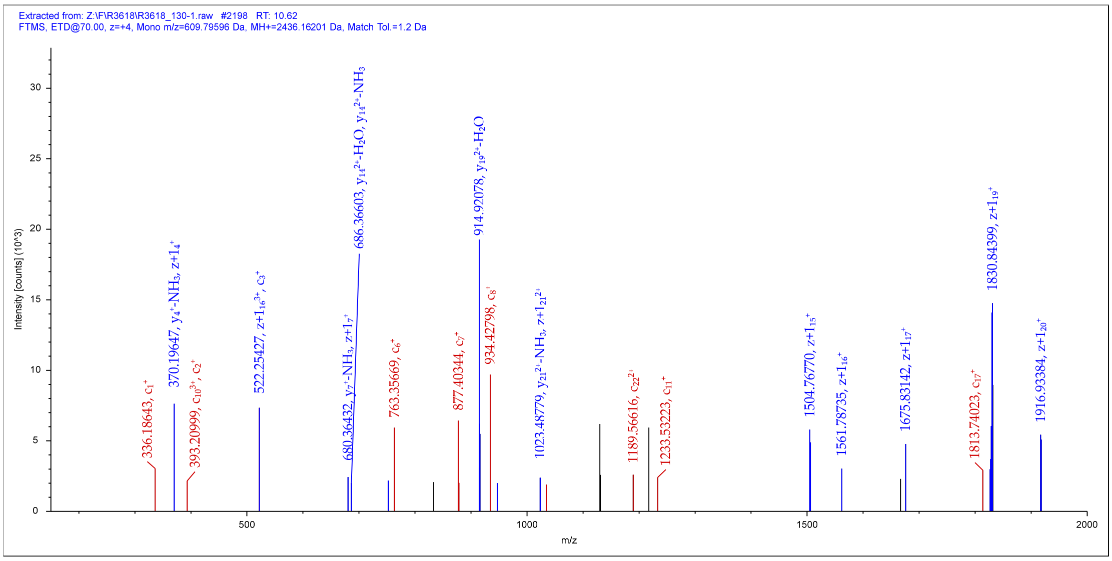

(B) KAFGHENEALVR**K**

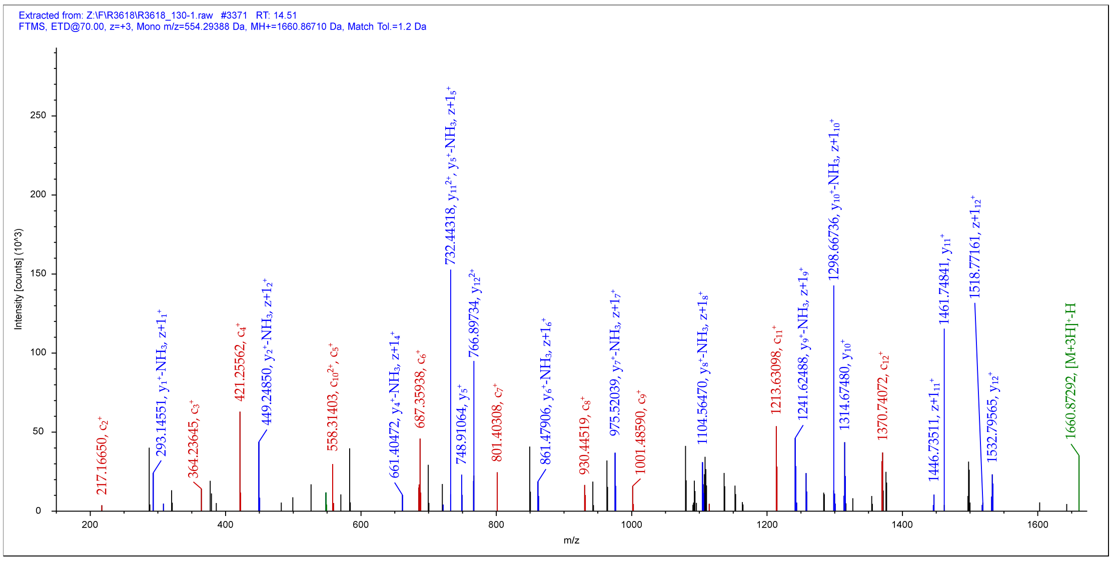

(C) G**R**PGEEGQPGAPGHQGPLGPR

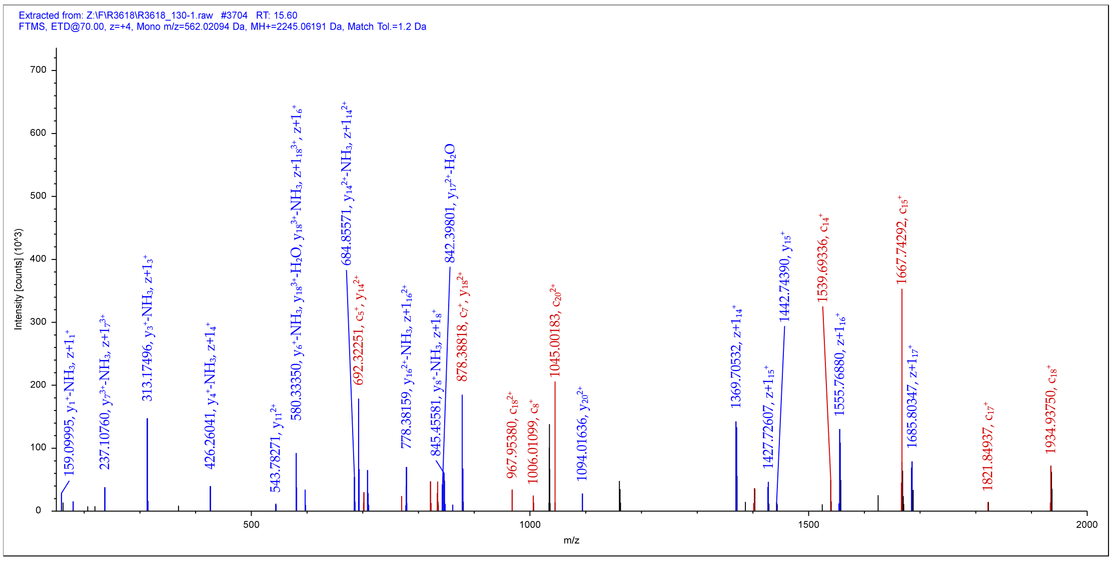

(D) DKDGKGKIPEEY

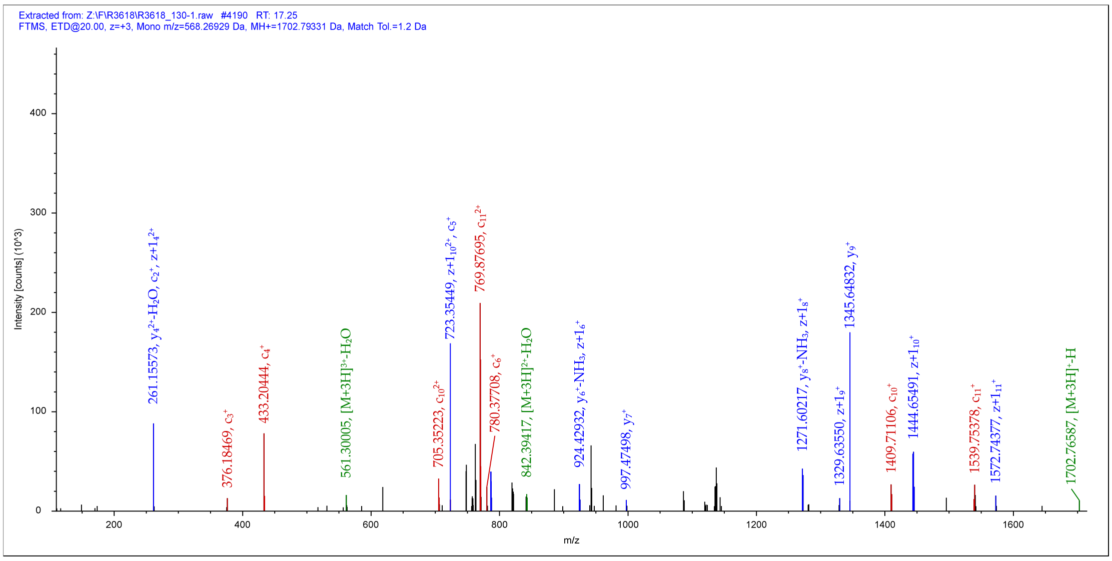

(E) DKGNKGTIPVED

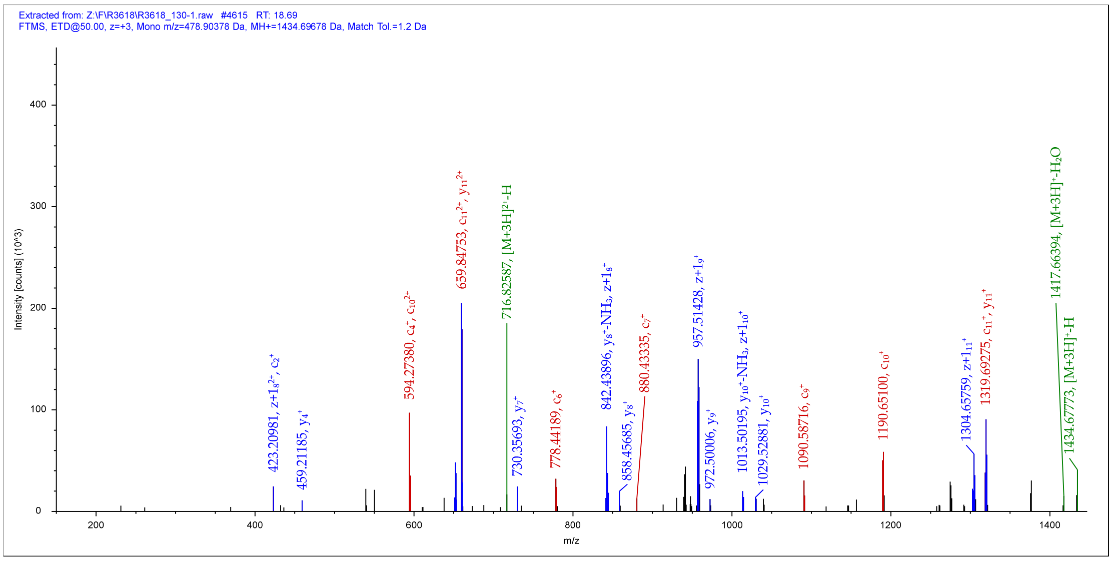

(F) DSRAATSPGELGVTIEGP**K**E

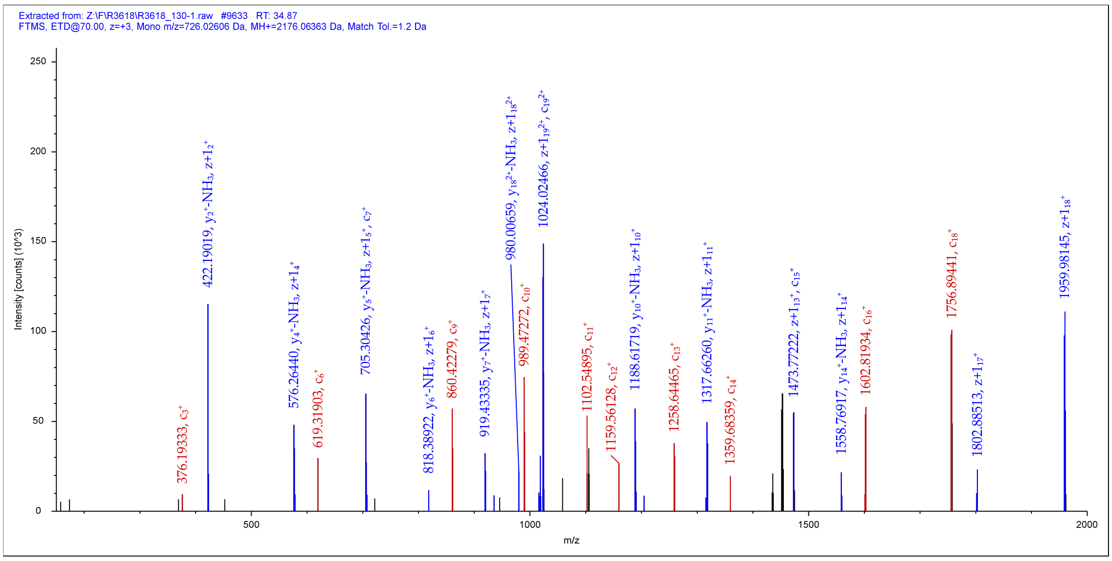

(G) DIVSEWV**K**FVTEEDSSKK

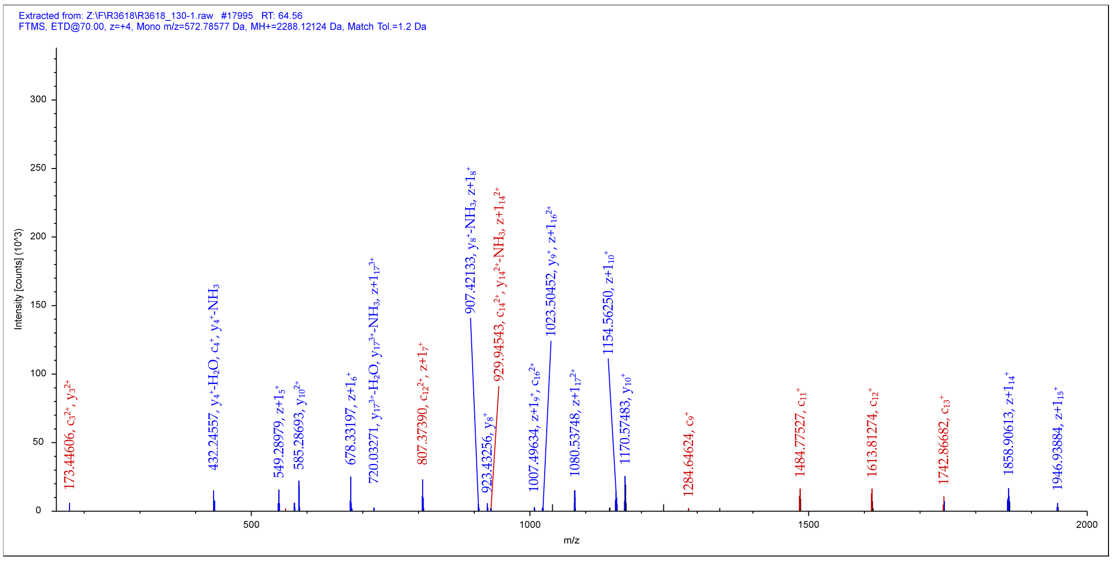

Fig. S5. (A-G) Annotated MS/MS spectra of identified glycosylated peptides from the 130-1 group.

130-3:

(A) <u>**R**</u>GESGPNGEPGRTGPPGPRGPRG

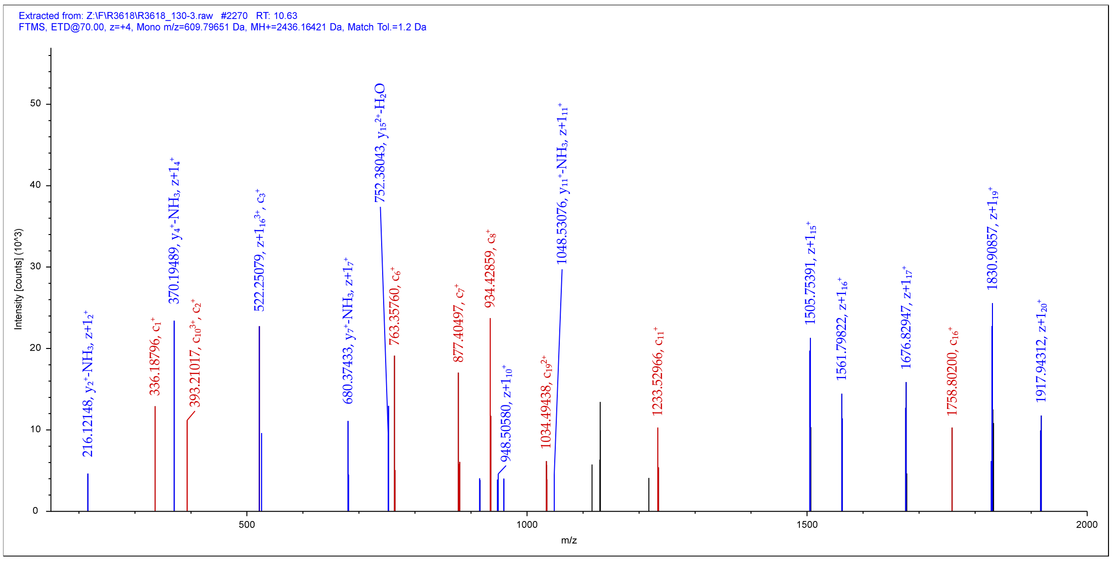

(B) SRN<u>**K**</u>FTNLH

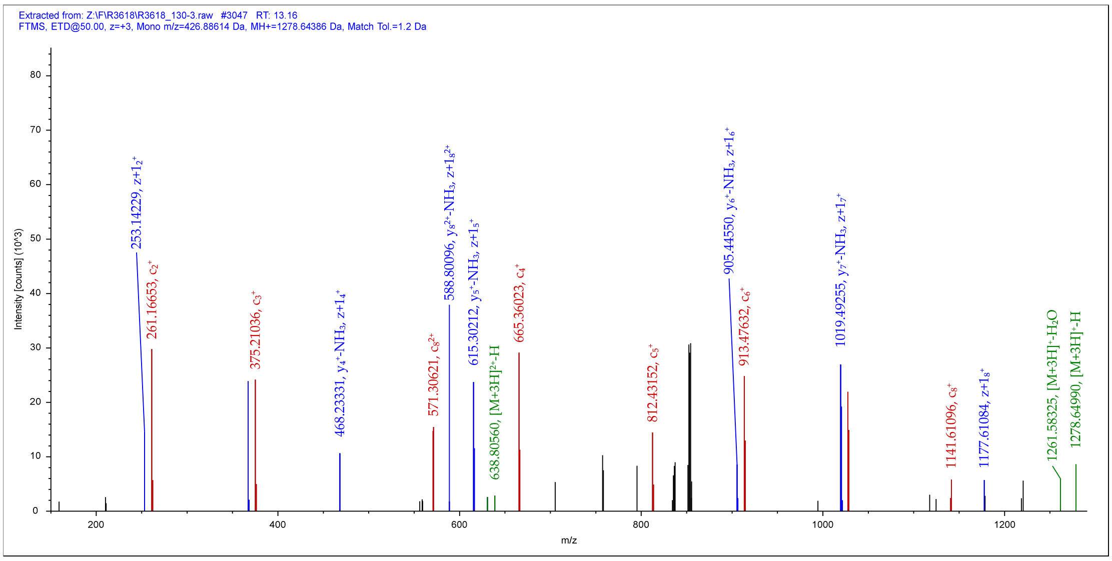

(C) G<u>**R**</u>PGEEGQPGAPGHQGPLGPR

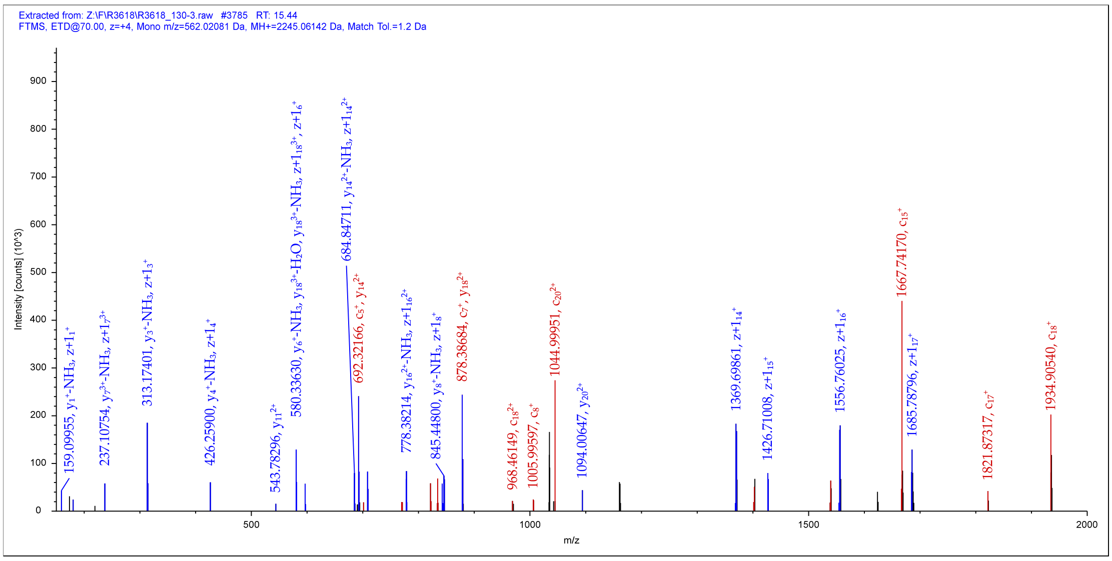

(D) AIRAGYDINK**K**A

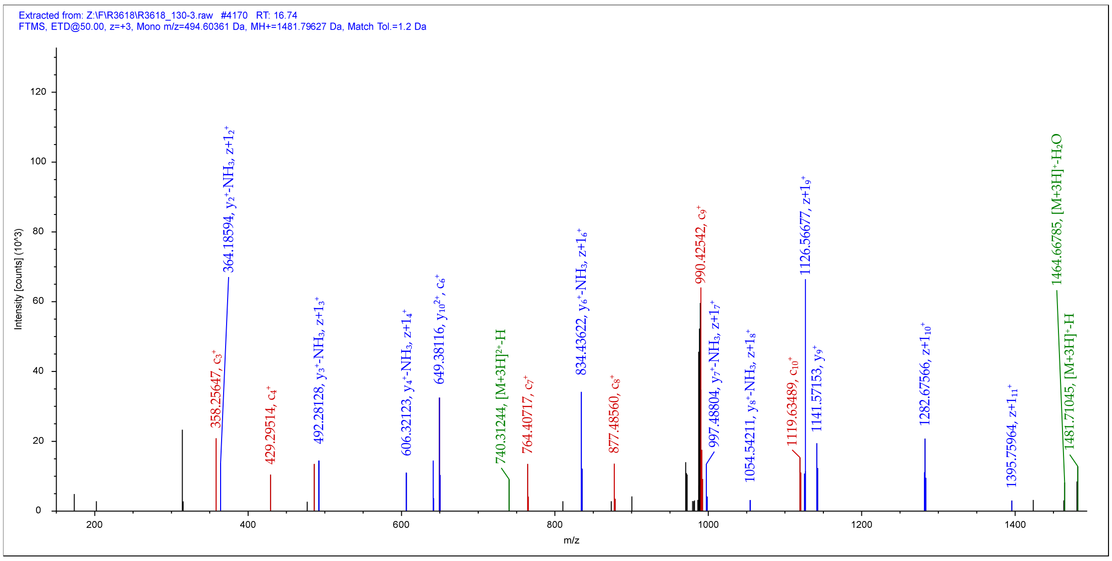

(E) D**K**DGKGKIPEEY

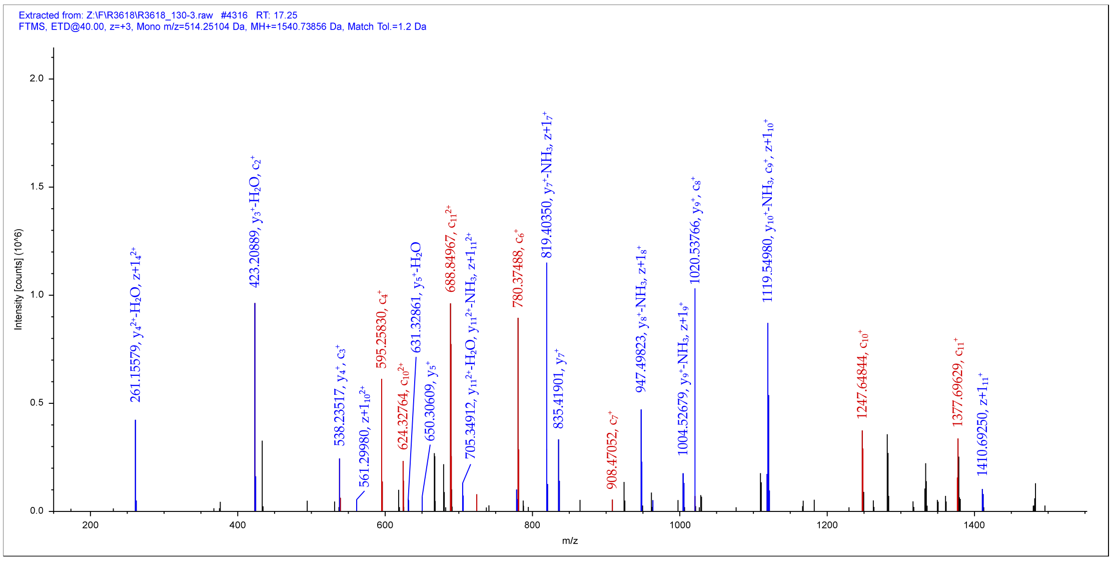

(F) A**K**IETKQNPDGTVGVT

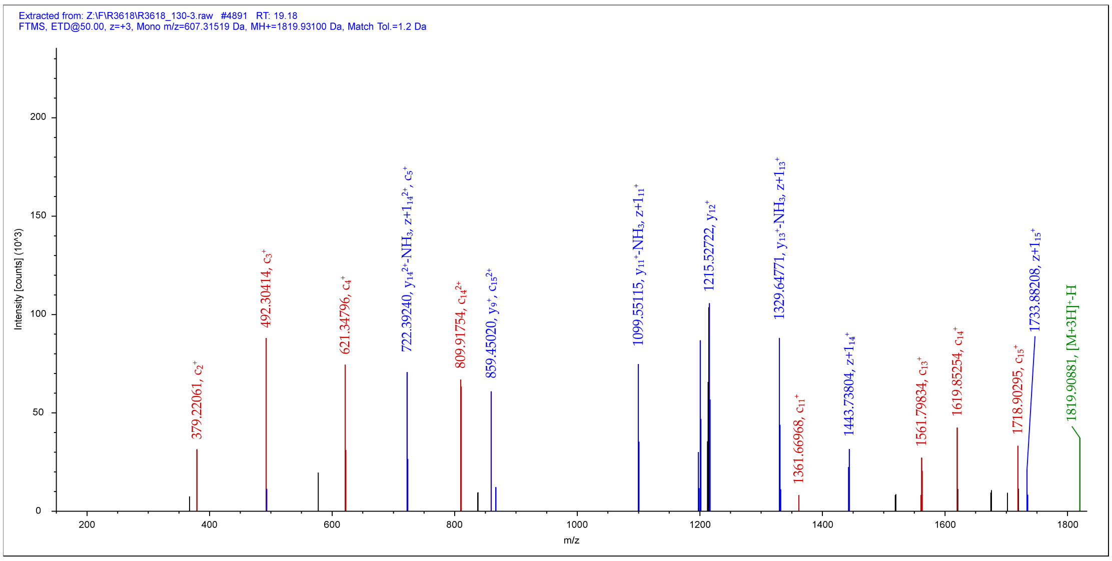

(G) DVIDTN**K**DRTIDE

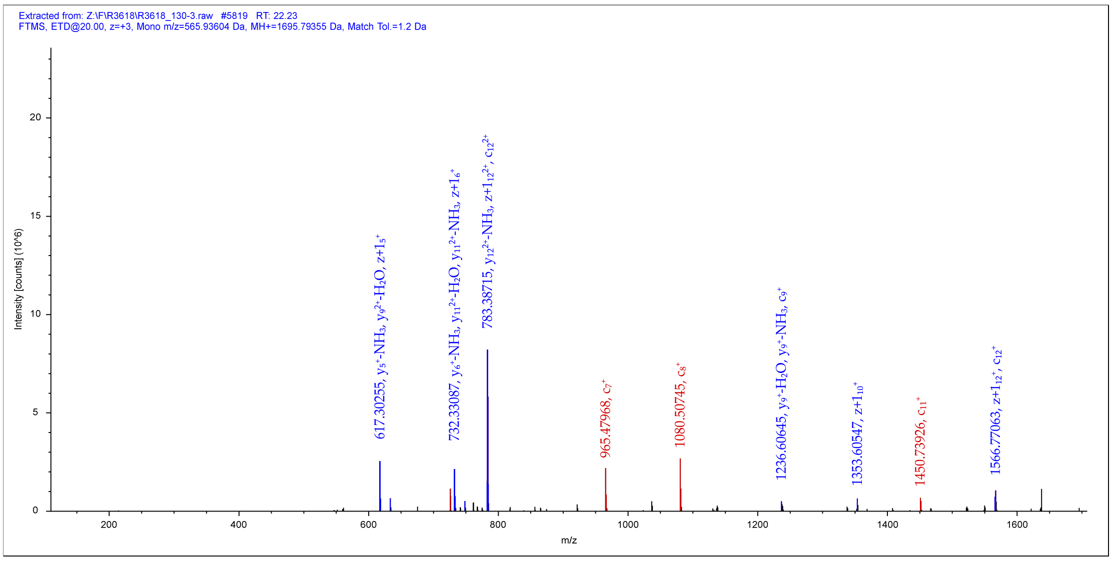

(H) VTVEGPSKV**K**L

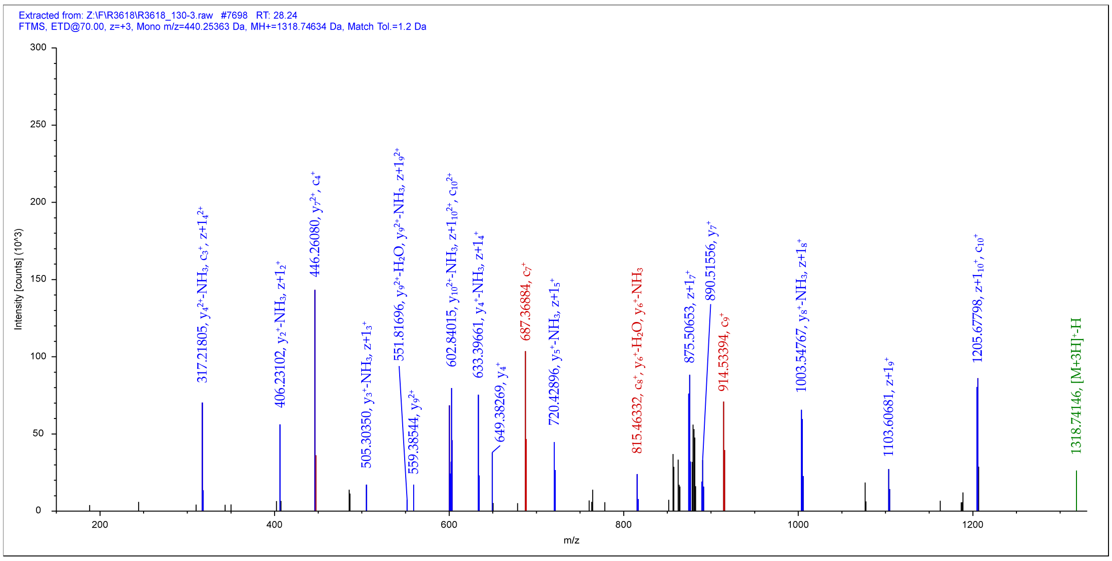

(I) SPF**K**VEVGPAKT

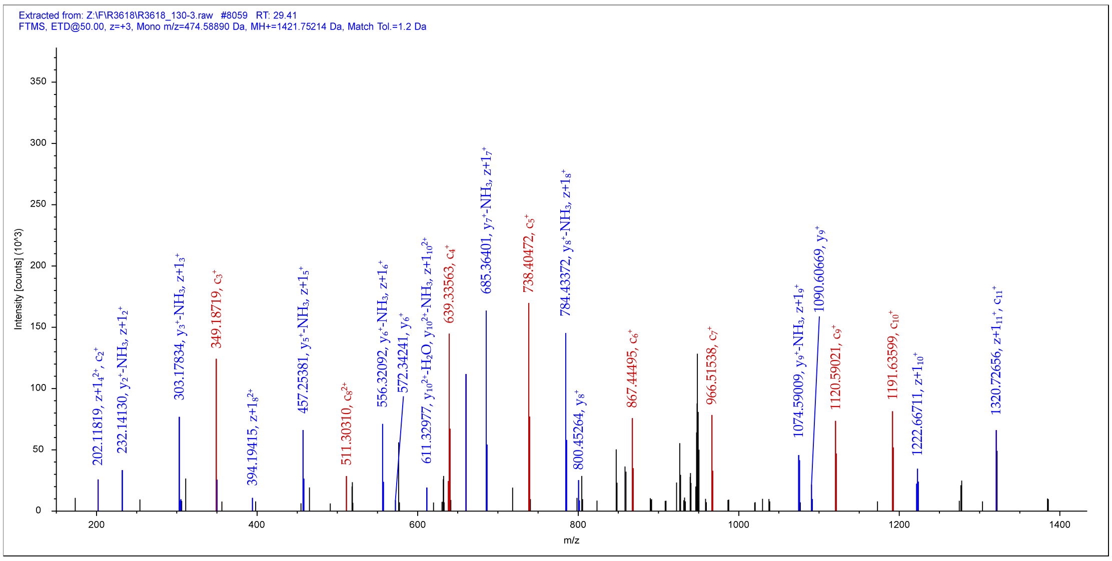

(J) DSRAATSPGELGVTIEGP**K**E

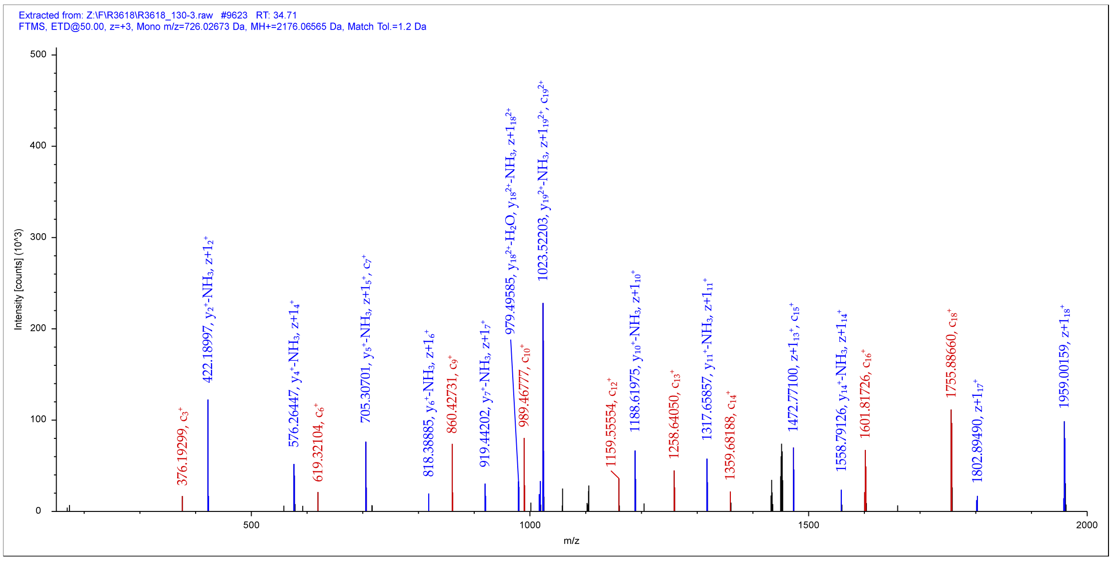

Fig. S6. (A-J) Annotated MS/MS spectra of identified glycosylated peptides from the 130-3 group.

130-5:

(A) RLPG**K**KKPR

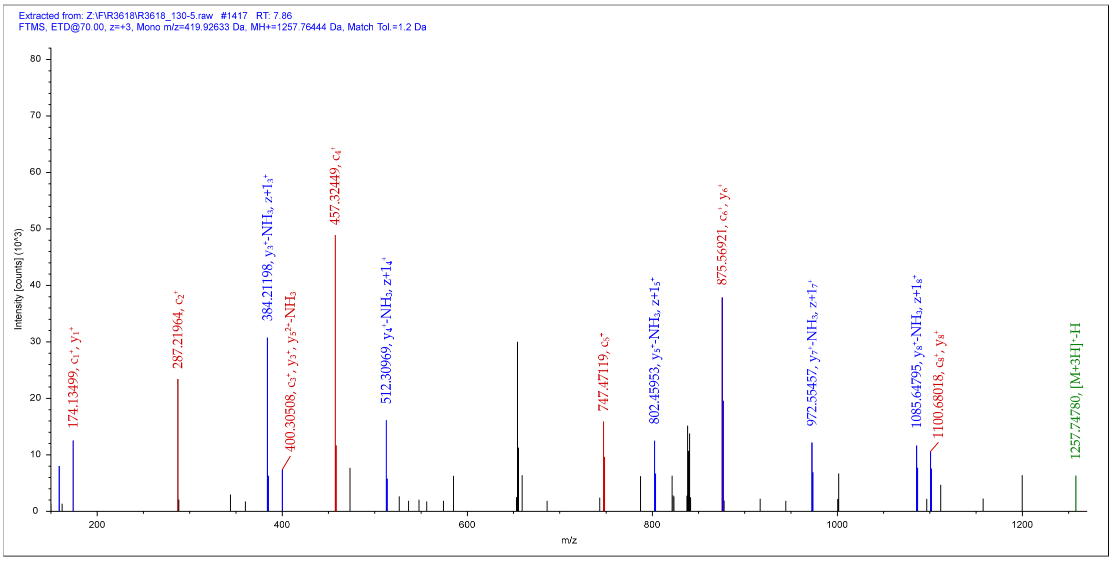

(B) GQ**R**GIPGERGRDGDRGSNG

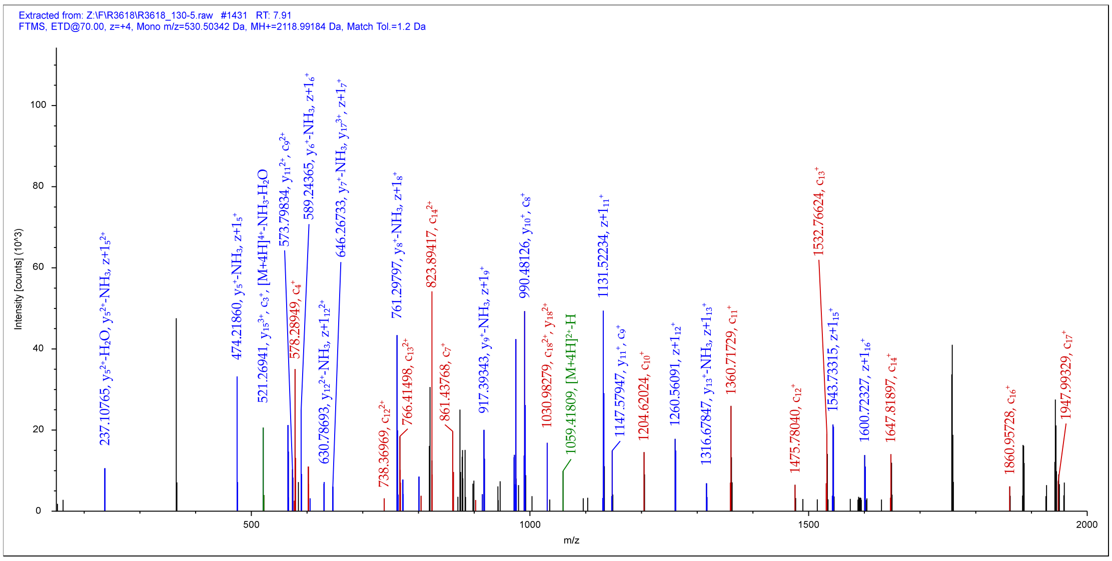

(C) APPVEEGGGK**K**

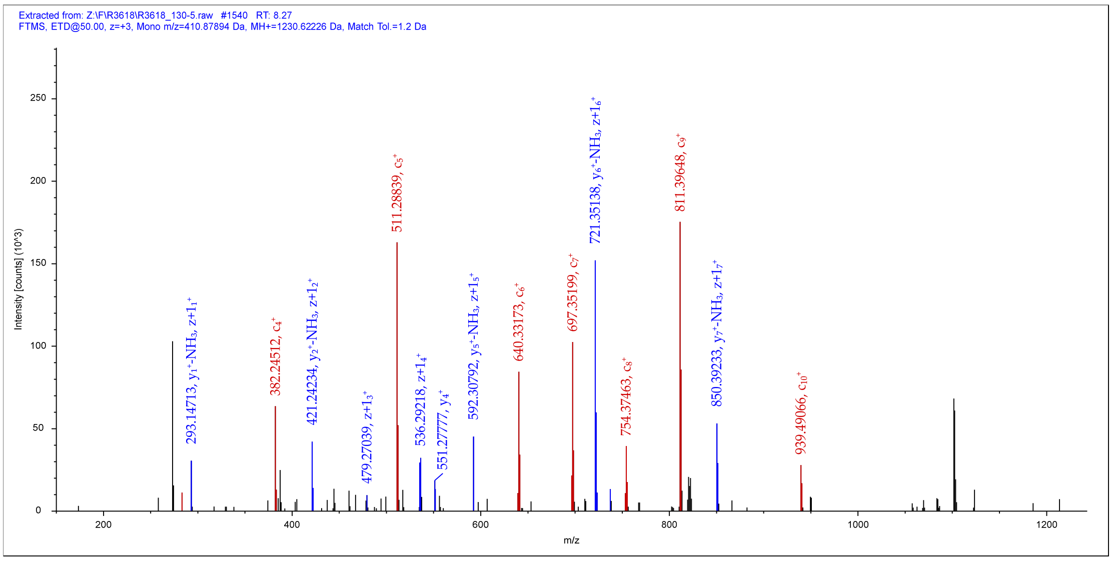

(D) <u>**R**</u>RGESGPNGEPGRTGPPGPRGPRG

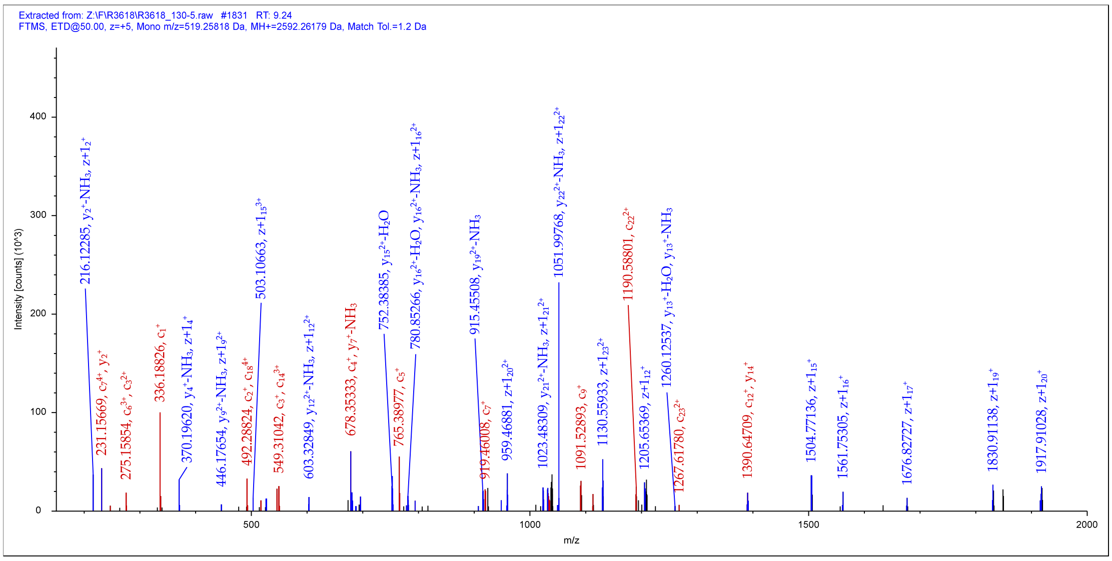

(E) GESGLPG<u>**R**</u>DGDSGPPGRQGGRG

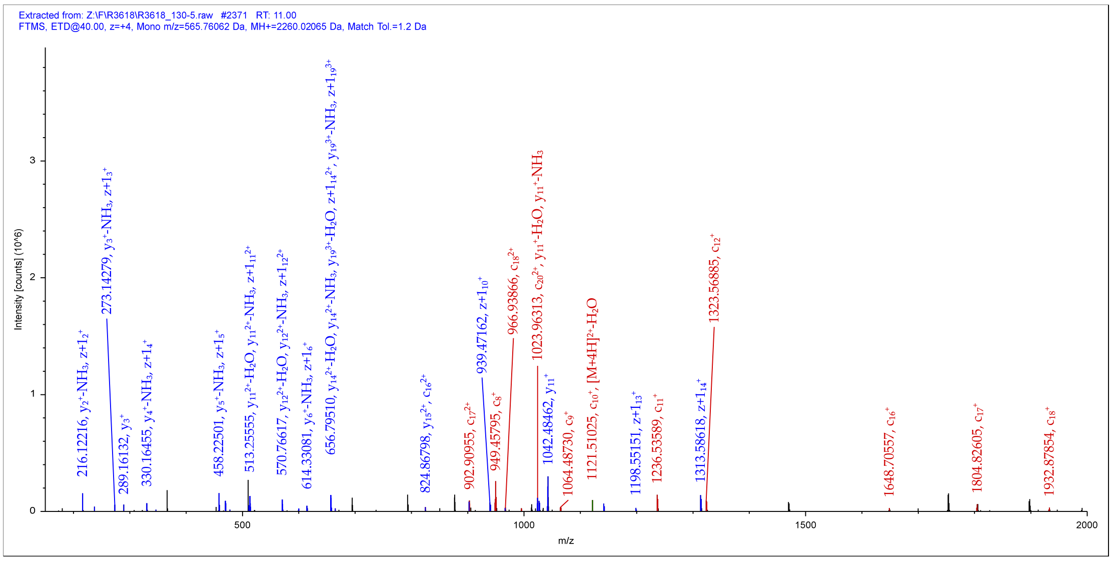

(F) SRN**K**FTNLH

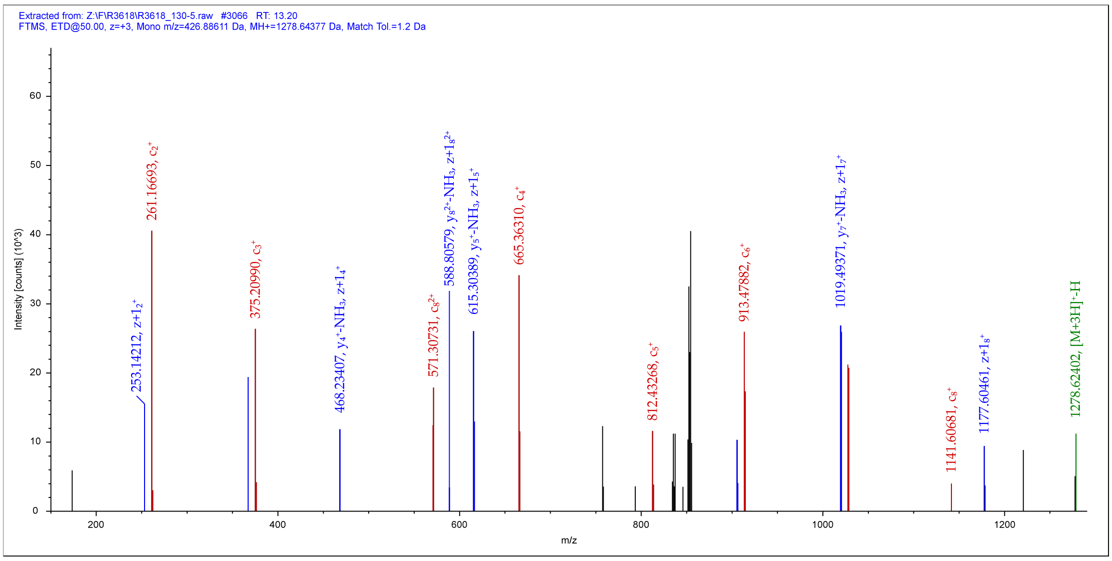

(G) G**R**PGEEGQPGAPGHQGPLGPR

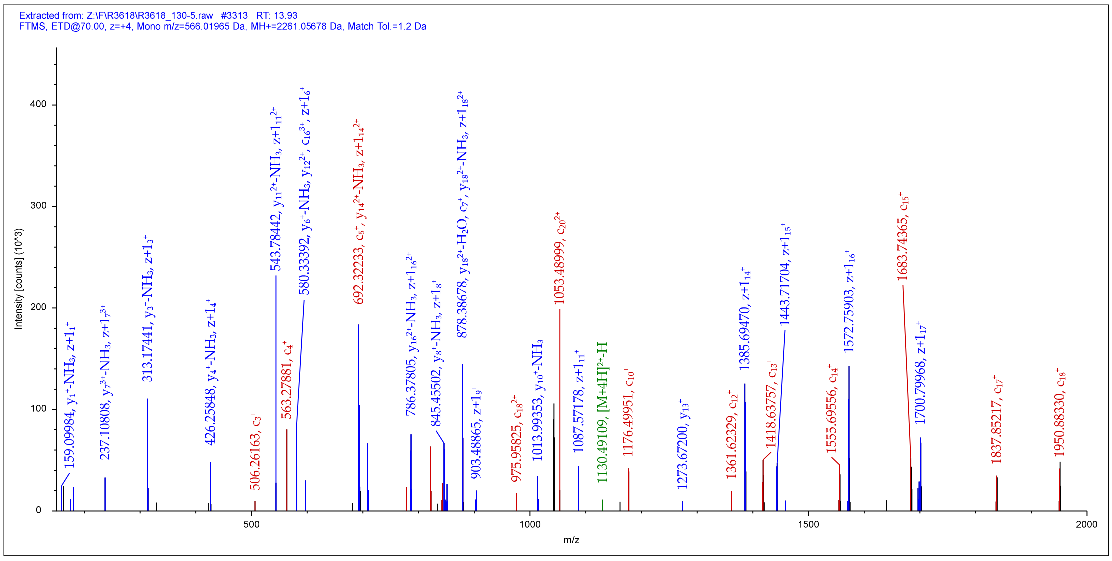

(H) G**K**DGPAGEHGSPGPLGPR

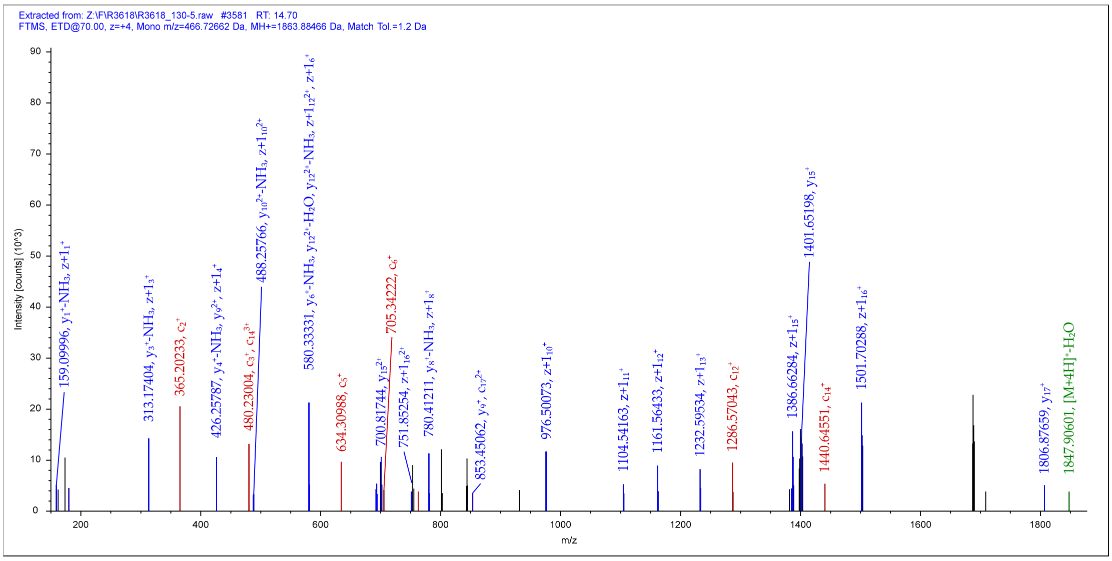

(I) YISLEELYKIMTT**K**

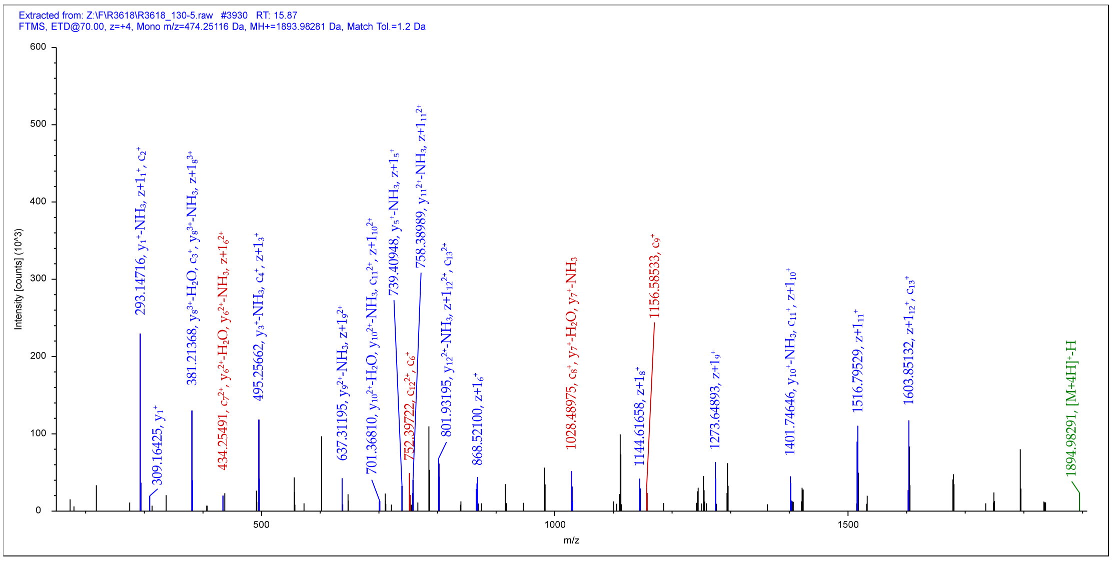

(J) SLYNKENKHVPL**K**

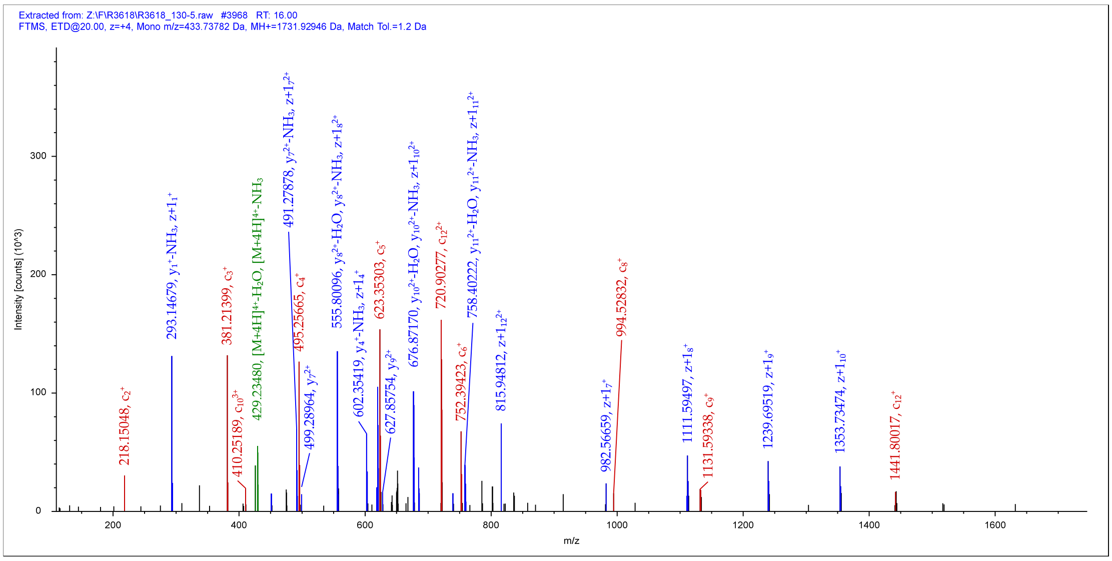

(K) D**K**DGKGKIPEEY

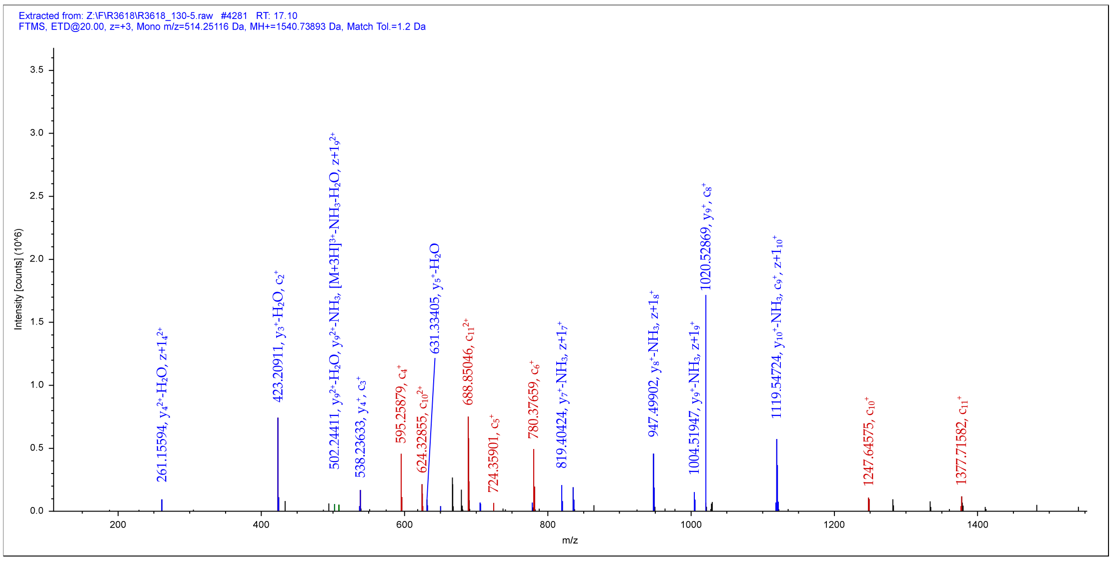

(L) NLHELVGD**K**AKGVQVNF

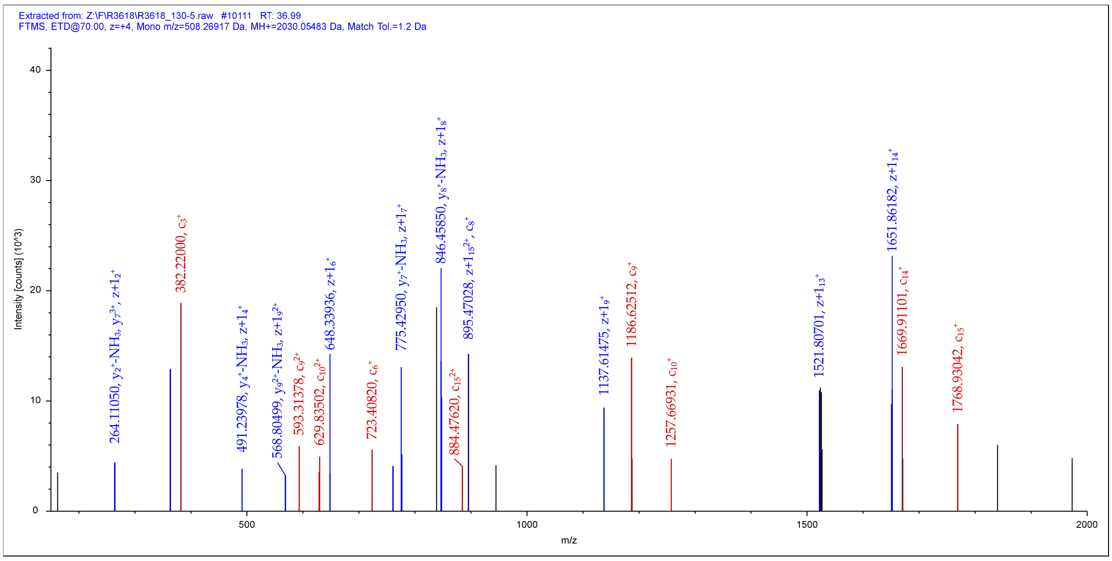

(M) DEFVYAF**K**AFGHEN

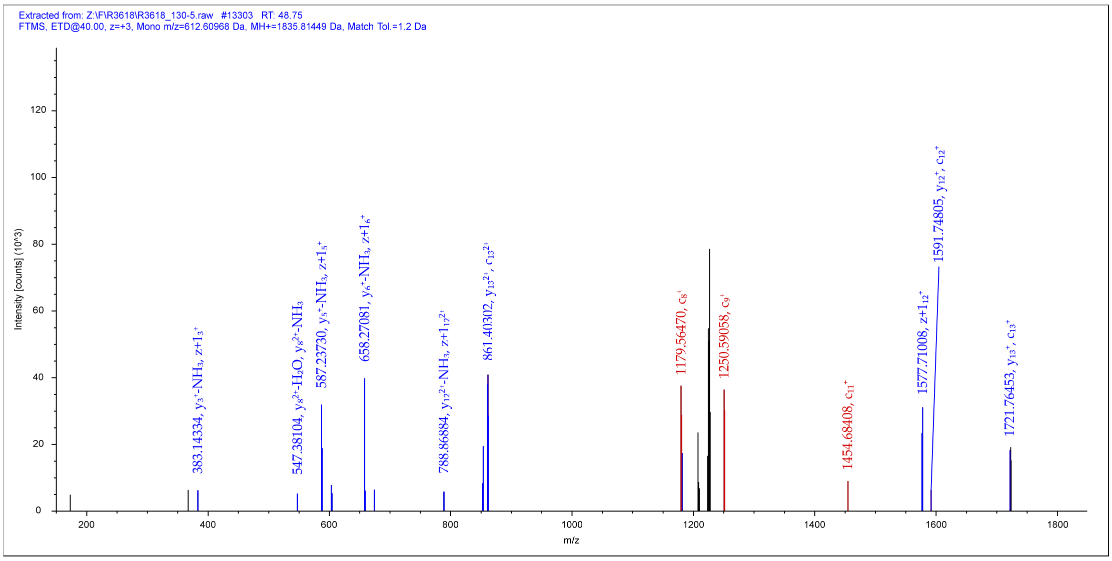

(N) DIVSEWV**K**FVTEEDSS**K**K

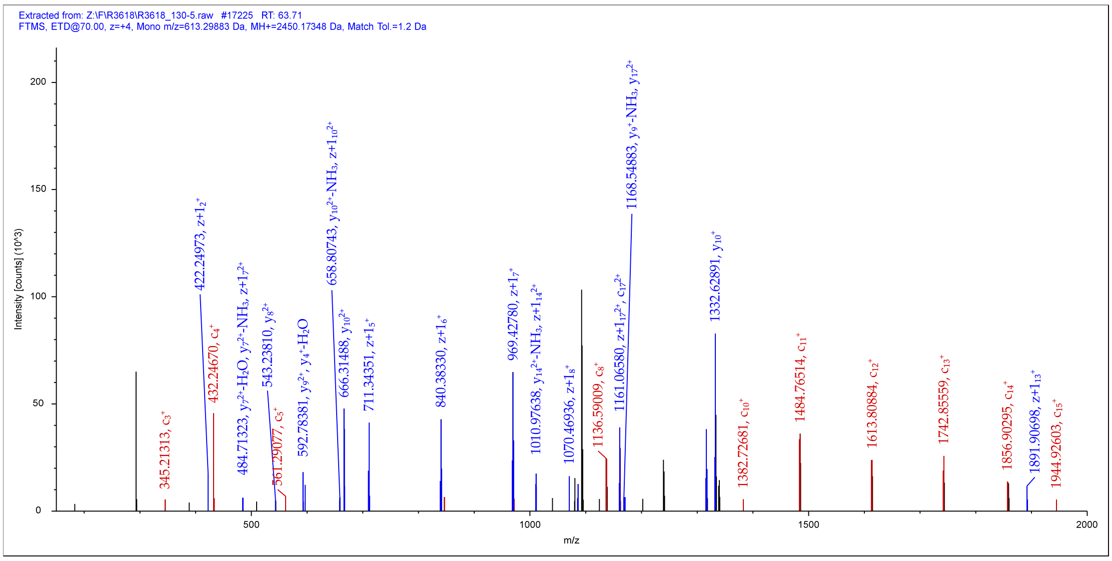

Fig. S7. (A-N) Annotated MS/MS spectra of identified glycosylated peptides from the 130-5 group.
